# Activated factor X targeted stored in platelets as an effective gene therapy strategy for both hemophilia A and B

**DOI:** 10.1002/ctm2.375

**Published:** 2021-03-24

**Authors:** Dawei Wang, Xiaohu Shao, Qiang Wang, Xiaohong Pan, Yujun Dai, Shuxian Yao, Tong Yin, Zhugang Wang, Jiang Zhu, Xiaodong Xi, Zhu Chen, Saijuan Chen, Guowei Zhang

**Affiliations:** ^1^ State Key Laboratory of Medical Genomics, Shanghai Institute of Hematology, Rui Jin Hospital Affiliated to Shanghai Jiao Tong University (SJTU) School of Medicine Key Laboratory of Systems Biomedicine of Ministry of Education, Shanghai Center for Systems Biomedicine SJTU Shanghai China; ^2^ National Research Center for Translational Medicine Ruijin Hospital Affiliated to Shanghai Jiao Tong University School of Medicine Shanghai China; ^3^ Key Laboratory of Aging and Cancer Biology of Zhejiang Province Department of Basic Medical Sciences Hangzhou Normal University School of Medicine Hangzhou Zhejiang Province China; ^4^ Shanghai Research Center for Model Organisms Shanghai China

**Keywords:** factor X, gene therapy, hemophilia, inhibitor, platelet

## Abstract

**Background:**

Treatment of hemophiliacs with inhibitors remains challenging, and new treatments are in urgent need. Coagulation factor X plays a critical role in the downstream of blood coagulation cascade, which could serve as a bypassing agent for hemophilia therapy. Base on platelet‐targeted gene therapy for hemophilia by our and other groups, we hypothesized that activated factor X (FXa) targeted stored in platelets might be effective in treating hemophilia A (HA) and B (HB) with or without inhibitors.

**Methods:**

To achieve the storage of FXa in platelets, we constructed a FXa precursor and used the integrin αIIb promoter to control the targeted expression of FXa precursor in platelets. The expression cassette (2bFXa) was carried by lentivirus and introduced into mouse hematopoietic stem and progenitor cells (HSPCs), which were then transplanted into HA and HB mice. FXa expression and storage in platelets was examined in vitro and in vivo. We evaluated the therapeutic efficacy of platelet‐stored FXa by tail bleeding assays and the thrombelastography. In addition, thrombotic risk was assessed in the recipient mice and the lipopolysaccharide induced inflammation mice.

**Results:**

By transplanting 2bFXa lentivirus‐transduced HSPCs into HA and HB mice, FXa was observed stably stored in platelet α‐granules, the stored FXa is releasable and functional upon platelet activation. The platelet‐stored FXa can significantly ameliorate bleeding phenotype in HA and HB mice as well as the mice with inhibitors. Meanwhile, no FXa leakage in plasma and no signs of increased risk of hypercoagulability were found in transplantation recipients and lipopolysaccharide induced septicemia recipients.

**Conclusions:**

Our proof‐of‐principle data indicated that target expression of the FXa precursor to platelets can generate a storage pool of FXa in platelet α‐granules, the platelet‐stored FXa is effective in treating HA and HB with inhibitors, suggesting that this could be a novel choice for hemophilia patients with inhibitors.

## Graphical abstract

The platelet‐delivered FXa is effective in ameliorating the hemorrhagic phenotype of both HA and HB murine models, even in the presence of inhibitors.

No sign of hypercoagulability was found in platelet FXa‐containing mice under either steady or pathological status.

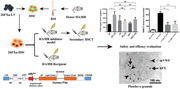

**Abbreviations**
AAV: adenovirus‐associated virus
ANOVA: one‐way analysis of variance
FIX: factor IX
FVa: activated factro V
FVIII: factor VIII
FXa: activated factor X
HA: hemophilia A
HB: hemophilia B
HSCT: hematopoietic stem and progenitor cell transplantation
HSPC: hematopoietic stem and progenitor cell
ITI: immune tolerance induction
LPS: lipopolysaccharide
NHP: normal human plasma
rhFIX: recombinant human FIX
rhFVIIa: activated
rhFVIII: recombinant human FVIII
RKR: Arg‐Lys‐Arg
RT: room temperature
RVV: russell's viper venom
SAP: saporin
TAT: thrombin‐antithrombin III complexes
TEG: thrombelastography
TF: tissue factor
VWF: von Willebrand factor
WBC: white blood cells
WBCT: whole blood clotting time

## 1 INTRODUCTION

Hemophilia A (HA) and hemophilia B (HB) are serious X‐linked bleeding diseases that mostly affect males, with a prevalence rate of 1 in 5,000 for the former and 1 in 30,000 for the latter.^1^ HA and HB are caused by defect or decrement of factor VIII (FVIII) or factor IX (FIX) in the blood, respectively. Currently, the common treatment of these diseases is replacement therapy. Patients with hemophilia can significantly benefit from prophylaxis at an early age but have to receive frequent injections to prevent bleeding.^2^ However, only 25% of hemophilia patients worldwide received efficient treatment and prophylactic therapy whereas many patients are unable to afford costs for prophylaxis.^3^ Moreover, up to 30% of patients with HA and 5% of patients with HB could develop inhibitors after replacement therapy. In these cases, the dosage of FVIII or FIX administration should be increased to retain therapeutic effects. If a high inhibitor titer is developed, the factors become ineffective and alternative treatments should be adopted.^4^ Patients with high titer inhibitors are mainly treated by the use of bypassing agents and immune tolerance induction (ITI). The currently available bypassing agents include recombinant human factor VIIa (rhFVIIa) and activated prothrombin complex concentrates, which are effective in approximately 80% of patients with hemophilia.^5^ ITI is proven to be effective in only 70% of patients with HA and 30% of patients with HB. Thus, a substantial proportion of patients cannot be successfully treated by current bypassing agents or ITI.^6^ Although prophylaxis using bypassing agents is effective in many patients, the efficacy is not as good as that of replacement factors. Moreover, the cost and adverse effects of bypassing agents remain the main clinical concerns.^7^

Factor X (FX) is critical in both the intrinsic and extrinsic pathways of the coagulation cascade. FX is activated by either FIXa/FVIIIa or FVIIa/tissue factor (TF), while the activated FX (FXa) converts prothrombin into thrombin to achieve hemostasis.^8^ Theoretically, as a factor that is downstream of FVIII and FIX, FXa could start coagulation without FVIII or FIX, constituting an ideal target of bypassing therapy. In the past decades, FXa was studied as a bypassing agent for the treatment of hemophilia.^9^, ^10^, ^11^ FXa exhibited a hemostatic effect on hemophilia upon direct infusion into plasma but had a very short half‐life.^12^ On the other hand, increased FXa concentration in plasma could cause disseminated intravascular coagulation.^9^ These limitations have hindered the application of FXa as a bypassing agent. Recently, Ivanciu et al developed a zymogen‐like FXa, which was inactive in the plasma but could be activated upon the combination with activated factor V (FVa).^13^ This new variant was proven effective in hemophilia mouse models and did not cause excessive coagulation. However, the half‐life of the zymogen‐like FXa was still not long enough to be used in prophylaxis.^14^ These results imply that albeit some limitations, FXa could still be a potential bypassing agent for the treatment of hemophilia upon innovation.

Over the past three decades, gene therapy has become a field of intensive research for the treatment of genetic diseases including hemophilia. Previous studies proved that platelets could be an effective drug delivery system, expressing coagulation factors ectopically in platelets through gene transfer could store proteins in platelets and these factors could be released at injury sites to accelerate coagulation.^15^, ^16^, ^17^ We thus hypothesized that if FXa could be stored in platelets and released during platelet activation, then it could be released and promote hemostasis primarily at the injury site. At the same time, the exposure of FXa in plasma could be limited, so that the prothrombotic risk of FXa might be controlled and rapid clearance of FXa be avoided. In this scenario, the platelet‐stored FXa would be an ideal FXa source and could be used to treat hemophilia patients, particularly those who developed inhibitors.

In the present study, we placed an FXa precursor under control of the integrin αIIb promoter, a megakaryocyte/platelet‐specific promoter, and performed proof of principle studies to demonstrate that FXa could be synthesized and stored in platelets through the tissue‐specific expression. We showed that the platelet‐stored FXa could be released upon platelet activation at injury sites, and ameliorate bleeding disorders of HA and HB mice, especially the mice developed FVIII and FIX inhibitors, respectively. No increasing thrombotic risks were observed in the mice.

## 2 MATERIALS AND METHODS

### 2.1 Vector construction

**FIGURE 1:**
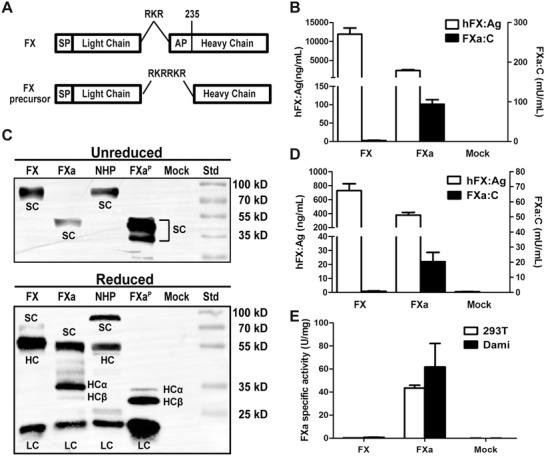
**FXa construction and in vitro expression**. (**A**) Schematic of FX and FXa precursor cassettes. For FX, a PACE/furin cleavage site RKR between the light chain and activation peptide (AP) and a zymogen cleavage site at Arg‐235 were denoted. For FXa precursor, AP was deleted and replaced by RKR. FX, normal human FX; FXa, factor Xa; SP, signal peptide. (**B**) Quantitative evaluation of hFXa:Ag and FXa:C levels in HEK293T cells (n = 3). The cells were transfected with pCIneoFX, and pCIneoFXa and cell lysates were collected for hFXa:Ag and FXa:C assays. (**C**) Western blotting analysis of FX and FXa expressed in 293T cells. The FX and FXa expression status were evaluated under reduced or unreduced conditions, normal human plasma (NHP), and FXa protein (FXa^P^) were loaded as a positive control, mock vector as a negative control. SC, single chain; HCα, 35 kDa FXaα HC; HCβ, 31 kDa FXaβ HC; LC, light chain; Std, standard molecular weight markers. (**D**) Quantitative evaluation of hFXa:Ag and FXa:C levels in Dami cells (n = 3). The cells were transfected with pCIneo2bFX and pCIneo2bFXa, and cell lysates were collected for hFXa:Ag and FXa:C assays. (**E**) The specific activity of FXa from HEK293T and Dami cells. Specific activity was calculated by dividing FXa:C by hFX:Ag. Data are representative of three independent experiments

The αIIb promoter and plasmids pWPT2bF9, pCMV R8.91, and VSV‐G were kindly provided by Dr. David A. Wilcox (BloodCenter of Wisconsin, WI, USA). Full‐length hFX cDNA was amplified from human fetal liver cDNA (Table S1) and cloned into plasmid pCIneo to generate the corresponding expression vector pCIneoFX. The FXa precursor, an hFX variant, was constructed from pCIneoFX by replacing the activation peptide of FX with the PACE/furin cleavage sequence Arg‐Lys‐Arg (RKR) as previously reported (Figure 1A),^18^ generating pCIneoFXa. The FX and FXa precursor cassettes were then subcloned into vector pCIneo2bF9 to place the target cassettes under control of the αIIb promoter,^17^ generating pCIneo2bFX and pCIneo2bFXa, respectively. The *2bFXa* in pCIneo2bFXa was subcloned into vector pWPT2bF9,^17^ to replace the *2bF9* fragment and generate pWPT2bFXa. All recombinant plasmids were confirmed by DNA sequencing.

A lentiviral vector carrying *2bFXa* (2bFXa‐LV) was produced by a calcium phosphate transient transfection method.^17^ Briefly, plasmids pWPT2bFXa, pCMV R8.91, and VSV‐G were co‐transfected into Lenti‐X 293T cell line (Takara, Mountain View, CA). The lentivirus was harvested 24, 48, and 72 h after transfection and resuspended in X‐vivo 15 medium (Lonza, Basel, Switzerland). The lentivirus was then stored at −80°C until use. The virus titer was measured through a real‐time PCR method.^17^

### 2.2 Animals

HA mice on S129 background were purchased from the Shanghai Research Center for Model Organisms (Shanghai, China). The mouse model was an *F8* Knockout mouse made by deleting exons 16−19 from the *F8* gene.^19^ All of these phenotypes were the same as the previously reported *F8* knockout mouse model.^19^, ^20^ HB mice on the C57BL/6 background were supplied by Jackson Laboratory (Bar Harbour, ME, USA). All mice were maintained in special pathogen‐free rooms and permitted for use by the Ethics Committee of Ruijin Hospital Affiliated to Shanghai Jiao Tong University School of Medicine. The mice used for experiments were 6−12 weeks old, and only male mice were chosen as recipients of hematopoietic stem and progenitor cell transplantation (HSCT).

### 2.3 FXa activity (FXa:C) assay

FXa:C was assessed by a modified chromogenic assay that we developed using an FX assay kit (HYPHEN BioMed, Neuville‐sur‐Oise, France). FXa:C was measured directly by an FXa specific substrate, SXa‐11, provided in the kit. Russell's viper venom (RVV) treated pooled normal human plasma (NHP) was used as a standard. Pooled NHP was prepared from 30 normal individuals. RVV was used to activate FX in plasma into FXa. We assumed that all FX in pooled NHP were activated into FXa by RVV so that the FXa level was defined as 1 U/mL. A standard curve of FXa was plotted and FXa:C for samples was calculated.

To perform the assay, standards were prepared by serial diluting in Tris‐NaCl buffer. Supernatants collected 48 h after transfection and mouse blood samples were diluted 1:10 in Tris‐NaCl buffer. Standards and samples were added into wells of a 96‐well plate at 50 μL/well. The plate was incubated at 37°C for 2 min, and 50 μL of SXa‐11 substrate preincubated at 37°C was added into each well. Subsequently, RVV was added to the standards at 50 μL/well to activate FX, and an equal volume of Tris‐NaCl buffer was added into wells of the sample. The plate was mixed for 10 s on an orbital shaker and incubated at 37°C for 5 min. Fifty microliters of citric acid (20 g/L) was added to each well to stop the reaction. The plate was read at 405 nm.

### 2.4 hFX antigen (hFX:Ag) assay

Antigen levels of FX and FXa were measured by an hFX specific ELISA. This assay cannot distinguish between FX and FXa. A 96‐well plate was coated with 1 μg/mL goat anti‐hFX polyclonal antibody (Affinity Biologicals, Ancaster, Canada) in NaHCO_3_ buffer (pH 9.6) at 4°C overnight. The coated plate was blocked by 5% BSA in Tris‐buffered saline (TBS) at RT for 2 h and washed three times with phosphate‐buffered saline (PBS). Both the samples and standards were diluted in a dilution buffer (0.1% BSA in TBS), added at 100 μL/well, and incubated at RT for 1 h. After washing at least three times, each well was added with HRP‐conjugated goat anti‐hFX polyclonal antibody (Affinity Biologicals) diluted at 1:5000 and incubated at RT for 1 h. The plate was washed at least five times. Each well was added with 100 μL of TMB substrate (Sigma) and incubated at RT for 20 min. The reaction was terminated by adding 50 μL of 2 M H_2_SO_4_. Absorbance was read at 450 nm with pooled NHP as standard. hFX:Ag in pooled NHP was taken as 10 μg/mL.

### 2.5 Measurement of thrombin‐antithrombin III complexes (TAT), fibrinogen, and D‐Dimer

Mouse blood was collected from retro‐orbital puncture and platelet‐poor plasma was prepared for TAT, fibrinogen, and D‐Dimer measurement. To avoid TF contamination, the first drop of blood was discarded. Commercially available kits were used to determine levels of TAT (Abcam, Cambridge, MA, USA) and fibrinogen (Assaypro, Charles, MO, USA) according to the manufacturer's guidance. D‐dimer levels were detected using a mouse D‐Dimer ELISA kit (Westang, Shanghai, China). Briefly, a 96‐well plate was pre‐coated with a mouse D‐Dimer monoclonal antibody, standards (supplied with the kit) and diluted plasma samples were added at 100 μL/well and incubated at 37°C for 40 min. After washing 4 times, a biotinylated anti‐D‐Dimer antibody was added and incubated at 37°C for 20 min. HRP‐conjugated Streptavidin was added and incubated at 37°C for 10 min. The color was developed by TMB substrate and its absorbance measured at 450 nm.

### 2.6 Fibrin deposition assay

Mice were primed intradermally with 5 μg lipopolysaccharide (LPS) (Sigma) or normal saline control (no‐priming) in the foot and challenged 24 h later by intravenous injection of 300 μg LPS. Blood and tissue samples were obtained 6 h after LPS challenge. Each group was composed of 3 mice. Anesthetized mice were perfused with 20 mL of PBS through the left ventricle. The liver was removed, fixed in 10% formalin, and embedded in paraffin. After rehydration, antigen retrieval, peroxidase blocking, and protein blocking, sections were incubated overnight with anti‐fibrinogen antibody (Abcam) in a humidified chamber. Goat anti‐rabbit IgG peroxidase (Servicebio, Wuhan, China) was used as the secondary antibody for 30 min. Antigen‐antibody complexes were visualized with DAB Peroxidase Substrate.

### 2.7 Confocal microscopy and immunoelectron microscopy analysis

Freshly isolated mouse platelets were cytospinned and immunofluorescently stained by using previously described methods.^17^ A goat anti‐hFX antibody (Affinity Biologicals, Ancaster, Canada) and a rabbit anti‐human von Willebrand factor (VWF) (DAKO, Glostrup Municipality, Denmark) were used as primary antibodies. Alexa Fluor 488‐donkey anti‐goat IgG antibody (Invitrogen) and Alexa Fluor 594‐donkey anti‐rabbit IgG antibody (Invitrogen) were used as secondary antibodies. The samples were analyzed with a confocal microsope (TCS SP8, Leica, Wetzlar, Germany) and a super‐resolution microscope (DeltaVision OMX, GE, Boston, MA, USA) for localization of FXa.

The isolated platelet pellets were fixed with 4% paraformaldehyde and 0.2% glutaraldehyde, then embedded in LR white resin. Ultrathin sections (90 nm) were collected on formvar coated copper grids. Sections were then incubated with a goat anti‐human FX polyclonal antibody (Affinity Biologicals, Ancaster, Canada), and a sheep anti‐human VWF polyclonal antibody (Abcam, Cambridge, MA, USA), which could also recognize mouse VWF. A rabbit anti‐goat colloidal gold probe (10 nm, Sigma) and a donkey anti‐sheep colloidal gold probe (6 nm, Abcam) were used as the secondary antibodies. The sections were observed in the Department of Electron Microscopy of Faculty of Basic Medicine, Shanghai Jiao Tong University (SJTU) School of Medicine, using an H‐7650 transmission electron microscope (HITACHI, Japan) operating at 80 kv. Platelets from HA mice were used as controls.

### 2.8 Flow cytometry analysis

Isolated platelets were fixed with 1% paraformaldehyde at 4°C for at least 2 h, permeabilized by 0.5% Triton X‐100 in 2% BSA‐PBS and blocked with 2.5% normal donkey serum at RT for 30 min. The cells were incubated with goat anti‐hFX antibody (Affinity Biologicals, Ancaster, Canada) at 5 μg/mL at RT for 1 h. Alexa Fluor 488‐donkey anti‐goat IgG antibody (Invitrogen) was used as a secondary antibody. After washing at least three times by PBS, the platelets were resuspended in 500 μL of staining buffer and proceed to flow cytometry analysis.

### 2.9 HSCT

Bone marrow cells were flushed from tibias and femora of adult HA or HB mice. Sca‐1^+^ cells were sorted by MACS columns using anti‐mouse Sca‐1 microbeads (Miltenyi Biotec, Bergisch Gladbach, Germany). The sorted Sca‐1^+^ bone marrow cells were cultured in X‐vivo 15 medium supplemented with 8 μg/mL polybrene (Sigma), 50 ng/mL SCF (Peprotech, Rocky Hill, NJ, USA), 10 ng/mL IL‐3 (Peprotech), and 10 ng/mL IL‐6 (Peprotech). The cells were infected with 2bFXa‐LV with MOI of 20 and centrifuged at 2,000 rpm for 2 h. The infections were repeated three times at 24 h intervals. Subsequently, 2 × 10^5^ infected Sca‐1^+^ cells and 1 × 10^6^ Sca‐1^–^ cells were transplanted into 8−12 week old lethally irradiated (two doses of 5 Gy with a 4 h interval) hemophilia male mice through tail vein injection. For secondary HSCT, BM mononuclear cells (2 × 10^6^ cells/mouse) from the HA recipient mice 8 weeks after first HSCT were transplanted into lethally irradiated HA mice which developoed FVIII inhibitors. Tail vein blood was collected 4 weeks after HSCT for analysis.

### 2.10 Quantitative real‐time PCR of *2bFXa*

Peripheral Genomic DNA was extracted from white blood cells (WBC), and the *2bFXa* copy number was measured using a real‐time PCR method. The primers and probes of WPRE and endogenous albumin gene were listed in table S1. *2bFXa* copy number in cells was calculated by normalizing the quantity of WPRE to albumin gene. DNA from HA and HB mice were used as a negative control.

### 2.11 Mouse immunization to FVIII and FIX

HA or HB mice were immunized with 200 μL of recombinant human FVIII (rhFVIII, Baxter, Deerfield, IL, USA; 500 U/kg) or recombinant human FVIII (rhFIX, Pfizer, New York, NY, USA; 200 U/kg) in the presence of complete adjuvant (Sigma) through intraperitoneal injection to induce anti‐hFVIII or anti‐hFIX antibodies, respectively. Another two injections were performed similarly to the first except using an incomplete adjuvant (Sigma) at 3‐week intervals. hFVIII and hFIX inhibitors were determined using a modified Bethesda assay.^21^ Briefly, sequential dilutions of mouse plasma were incubated with equal volumes of 1 U/mL rhFVIII or rhFIX at 37°C for 2 h. Residual FVIII:C and factor IX activity were measured through FVIII and FIX chromogenic assays (Hyphen BioMed, Neuville‐sur‐Oise, France), respectively.

### 2.12 Thrombelastography (TEG) assay

The assay was established and modified in our group.^19^ Briefly, blood was collected from the retro‐orbital and mixed with 3.8% sodium citrate at the ratio of 9:1 and transferred into the Kaolin tube. About 340 μL of mixed whole blood was added into the TEG disposable cup pre‐added with 20 μL of 0.2 M CaCl_2_. Clotting analysis was initiated at 37°C using TEG 5000 analyzers (Haemoscope). The reaction time (*R* value) was recorded as the whole blood clotting time (WBCT).

### 2.13 Tail bleeding assay and tail clipping test

The tail bleeding assay was modified based on previous work.^22^ Mouse tail was preheated 10 min in 37℃ saline after anesthetized with 2.5% avertin. Bleeding was initiated by cutting the tip of tail at the length of 2 mm, followed by submerging the tail in a tube with 2 ml saline at 37°C. The bleeding lasted for 15 min. Then blood loss was determined by measuring the hemoglobin concentration in the saline. 100 μL saline with bloodshed was added into 2.5 ml hemoglobin reagent (Jiancheng Bioengineering, Nanjing, China) for 5 min at RT, and OD values were measured at 540 nm on a Nanodrop 2000 photometer (Thermo Fisher Scientific, MA, USA).

The tail clipping test was carried out as described in the previous report.^15^ Briefly, the mouse tail tip was cut at 1.5 mm diameter, and its survival was monitored for 24 h. The mice were checked and evaluated 6 h after tail clipping, if a mouse is very weak with hypothermia and still bleeding, the mouse will be euthanized and recorded as failed the test. All animals were euthanized at the end of the test.

### 2.14 Statistical analysis

Statistical analysis was performed with GraphPad Prism 6. All results are shown as mean ± SD. *P* ≤ 0.05 was considered a significant difference and evaluated by two‐tailed Student's *t* test or a one‐way analysis of variance (ANOVA) followed by multiple comparisons test.

## 3 RESULTS

### 3.1 Validation of *in vitro* and *in vivo* expression of the human FXa gene construct

We constructed an FXa precursor by replacing the activation peptide of hFX with a PACE/furin cleavage site so that the light chain and heavy chain were linked together with two PACE/furin cleavage sites (Figure 1A). To verify if the FXa precursor could generate FXa after expression, we transfected the vector of FXa precursor into HEK293T cells and quantitated the antigen and activity levels of FXa. FXa antigen (hFX:Ag) was confirmed at the level of 2333.3 ± 215 ng/mL (Figure 1B). FXa activity (FXa:C) was also detected at the average level of 101.4 ± 12.1 mU/mL. On the contrary, the FXa:C of wild‐type (WT) FX control was undetectable, although the antigen level of FX was high (11900 ± 1652.3 ng/mL). The results indicated that FXa could be generated with the expression of FXa precursor. Besides, we confirmed the expression of single chain (SC), heavy chain (HC), and light chain (LC) of FXa by western blot analysis (Figure 1C). SC at 72 kD and 55 kD were found for FX and FXa under unreduced condition, respectively. There were one HC (55 kDa) in FX, two forms of HC (FXaα HC at 35 kDa and FXaβ HC at 31 kDa) in FXa and one LC (17 kDa) in either of the two proteins (Figure 1C).^18^, ^23^ There was mainly FXaα HC in the recombinant FXa but FXaβ HC in the plasma‐derived FXa control.

αIIb promoter is a megakaryocyte/platelet specific promoter demonstrated by previous work.^15^, ^24^, ^25^, ^26^ We tested the *2bFXa* cassette in Dami cells, a megakaryocytic cell line. Cell lysates were collected for FXa antigen and activity analyses. Similar to the results from HEK293T cells, hFX:Ag was detected in both FXa and FX groups (377.6 ± 67.9 ng/mL and 729 ± 171.1 ng/mL, respectively). FXa:C was detected only in FXa group (22.03 ± 11.4 mU/mL) (Figure 1D). Specific activity was calculated by dividing FXa:C by hFX:Ag, and the specific activities of FXa were 43.49 ± 4.17 mU/mg in HEK293T cells and 61.72 ± 35.4 mU/mg in Dami cells (Figure 1E). The results confirmed that functional FXa could be synthesized under control of the αIIb promoter. To determine FXa expression in an *in vivo* situation, we performed hydrodynamic tail vein injection. pCIneoFXa and pCIneoFX were injected into HA mice. At 24 h after injection, mouse plasma was collected for hFX:Ag and FXa:C measurements. In accordance with our *in vitro* results, hFX:Ag was detected in both FXa and FX groups (750 ± 540 ng/mL and 3850 ± 2770 ng/mL, respectively) (Figure S1A). Notably, FXa:C was detected only in the FXa group (67.2 ± 47.8 mU/mL) (Figure S1B). These results demonstrated that FXa could be synthesized in HA mice and exhibit coagulation activity.

### 3.2 Platelet‐targeted expression of FXa in HA recipient mice

To target FXa expression in platelets, we employed a hematopoietic stem and progenitor cell (HSPC)‐based *ex vivo* strategy. We purified Sca‐1^+^ cells from the bone marrow of HA donor mice and transduced the cells with 2bFXa‐LV. Subsequently, the transduced cells were transplanted into lethally irradiated HA recipient mice. All blood cell counts in the recipient mice recovered to the normal range 8 weeks after HSCT and these counts maintained throughout the study period (Figure S2). The body weights of these mice decreased slightly within the first 4 weeks after HSCT and gradually recovered to normal range.

**FIGURE 2:**
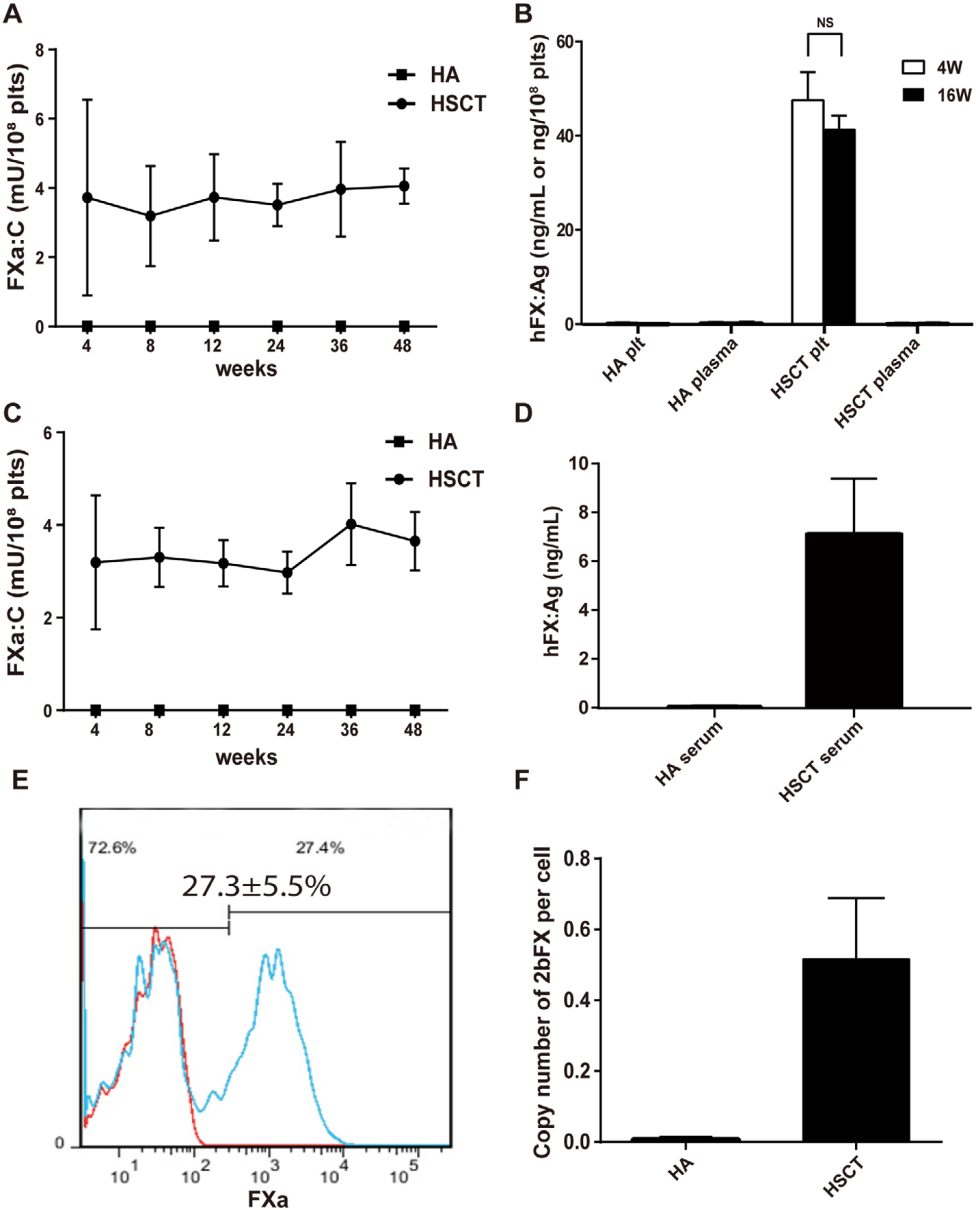
**Characterization of FXa in 2bFXa‐HSC‐transplanted HA mice**. (**A**) FXa:C levels in platelet lysates of transplantation recipients. HA, n = 3; HSCT, n = 7. FXa:C was measured from week 4 after HSCT throughout the study period. (**B**) Quantitative evaluation of hFX:Ag levels in mouse plasma and platelets. Mouse plasma and platelets were collected at week 4 and week 16. No hFX was detected in the plasma of transplantation recipients. HA, n = 3; HSCT, n = 7. (**C**) FXa:C level in platelet releasates of HA recipient mice. HA, n = 3; HSCT, n = 7. Mouse platelets were activated and releasates were collected for FXa:C assay. (**D**) hFX:Ag levels were detected in recipient mice serum immediately after the tail‐cutting. The hFX was released from activated platelets at injury site for coagulation. HA, n = 3; HSCT, n = 7. (**E**) Representative results of flow cytometry analysis of HA mouse platelets. The average number indicates the level of FXa positive platelets in 7 mice. Red line, HA mouse; blue line, transplantation recipient mouse. (**F**) *2bFXa* copy number in mice at 4 weeks after HSCT. HA, n = 3; HSCT, n = 7. A copy number of *2bFXa* in peripheral blood genomic DNA was measured. NS, not significant

A series of tail vein blood samples were collected from the recipients for multiple analyses started from week 4 after the transplantation. FXa was detected in platelet lysates. The average FXa:C was 3.72 ± 2.83 mU/10^8^ platelets at week 4 and remained steady until the end of the study at week 48 (Figure 2A). When median values according to the literature were taken, the average platelet count in the mice should be approximately 1 × 10^9^ platelets/mL. If the mouse blood volume is 2.5 mL,^27^ then the total platelet FXa in blood should be 93 mU. We assume plasma should account for half of the blood volume,^27^ the total plasma FX in a mouse should be 1.25 U and the platelet FXa:C should be 7.44% of the normal mouse plasma FX level. The hFX:Ag in platelets were 47.57 ± 18.05 ng/10^8^ platelets at week 4 and 41.28 ± 8.9 ng/10^8^ platelets at week 16 (Figure 2B) and the corresponding specific activities of FXa were 78.2 ± 5.94 U/mg and 77.2 ± 3.02 U/mg, respectively. No significant differences were found between the two time points regarding both of the hFX:Ag and specific activity levels of FXa. Meanwhile, we collected recipient mouse plasma and measured hFX:Ag through the hFX specific ELISA, which should not cross‐react with mouse FX. No hFX:Ag was detected in the plasma of all recipients at the two time points (week 4 and week 16) (Figure 2B). To examine whether FXa could be released from platelets, we isolated mouse platelets, stimulated them using platelet agonists, and collected the supernatants for FXa:C assay. The FXa:C was 3.19 ± 1.45 mU/10^8^ platelets at week 4, and the level remained throughout the study period (Figure 2C). Compared to the FXa level in platelet lysates, approximately 85.7% of FXa was released upon platelet activation. In addition, to evaluate the release of platelet‐stored FXa after injury, we collected serum and measured hFX:Ag, which was 7.15 ± 5.91 ng/mL, demonstrating that FXa was released during blood clotting (Figure 2D).

Mouse platelets were also collected for estimation of FXa expression using flow cytometry. Approximately 27.3% ± 5.5% (ranging from 20.1%−33.5%) platelets of HSCT recipient mice were stained positive for hFX, no positive staining was observed in platelets from HA mice (Figure 2E). The *2bFXa* gene copy number was estimated at 0.52 ± 0.17 copies per cell (Figure 2F). Throughout the duration of the study, we kept on monitoring blood cell counts in recipient mice. Four weeks after transplantation, the red blood cells and platelets returned to the normal range. Whereas the WBC count remained low and recovered to the normal range at 8 weeks (Figure S2). The cell numbers remained within the normal range until week 48, the end of the study. Hence, hematopoiesis was successfully restored in the recipients.

**FIGURE 3:**
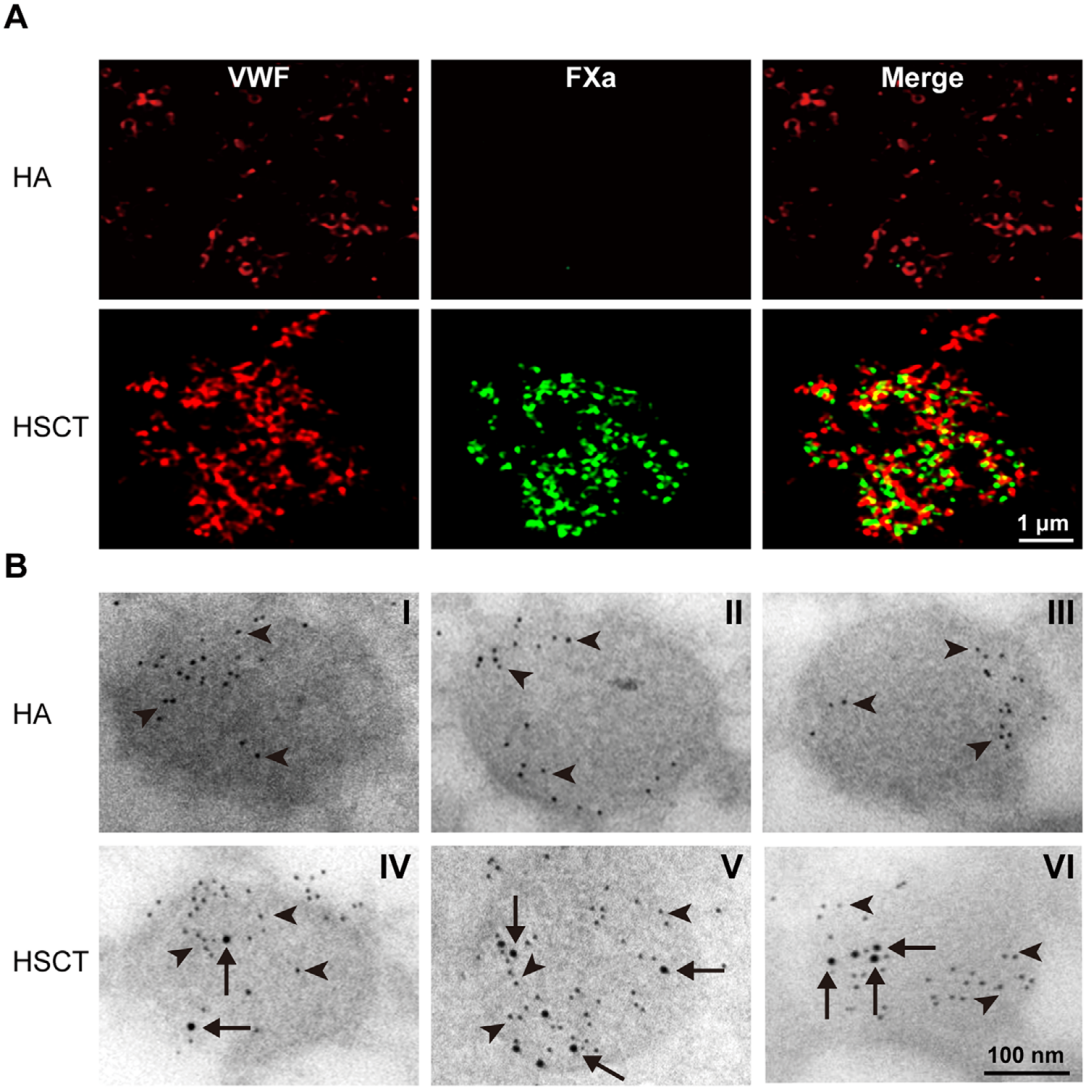
**Immunostaining of FXa**. Representative images of super‐resolution confocal microscopy analysis and immunoelectron microscopy analysis of FXa in platelets. (**A**) Super‐resolution confocal microscopy analysis of FXa in platelets. Platelets isolated from HA and HSCT recipient mice were immunostained for FXa (green) and VWF (red). The merged images show the proximity of FXa and VWF in the platelets of the HSCT recipient. Scale bars, 1 μm. (**B**) Electron microscopy determination of the cellular location of hFXa protein in platelets. Isolated platelets from untransplanted HA control mice (I‐III) and 2bFXa‐LV transduced HA recipients (IV‐VI) were immunostained for hFX (10 nm; some positive staining spots indicated by arrows) and endogenous mouse VWF (6 nm; some positive staining spots indicated by arrowheads). Three representative images were exhibited in each group. The results show that hFXa was localized in platelet α‐granules of 2bFXa‐LV transduced recipients. Scale bars, 100 nm

To confirm the storage of FXa in platelets, we collected platelets from HSCT recipient mice and subjected the cells to confocal microscopy analysis. FXa was demonstrated in platelets and colocalized with VWF, a marker of α‐granules of platelets (Figure S3). By using the super‐resolution microscopy analysis, FXa was observed near VWF (Figure 3A and Video S1). Finally, we demonstrated the localization of FXa in platelet α‐granules using immunoelectron microscopy (Figure 3B).

### 3.3 Phenotypic amelioration of HA recipient mice and evaluation of thrombotic risk of FXa‐based gene therapy

**FIGURE 4:**
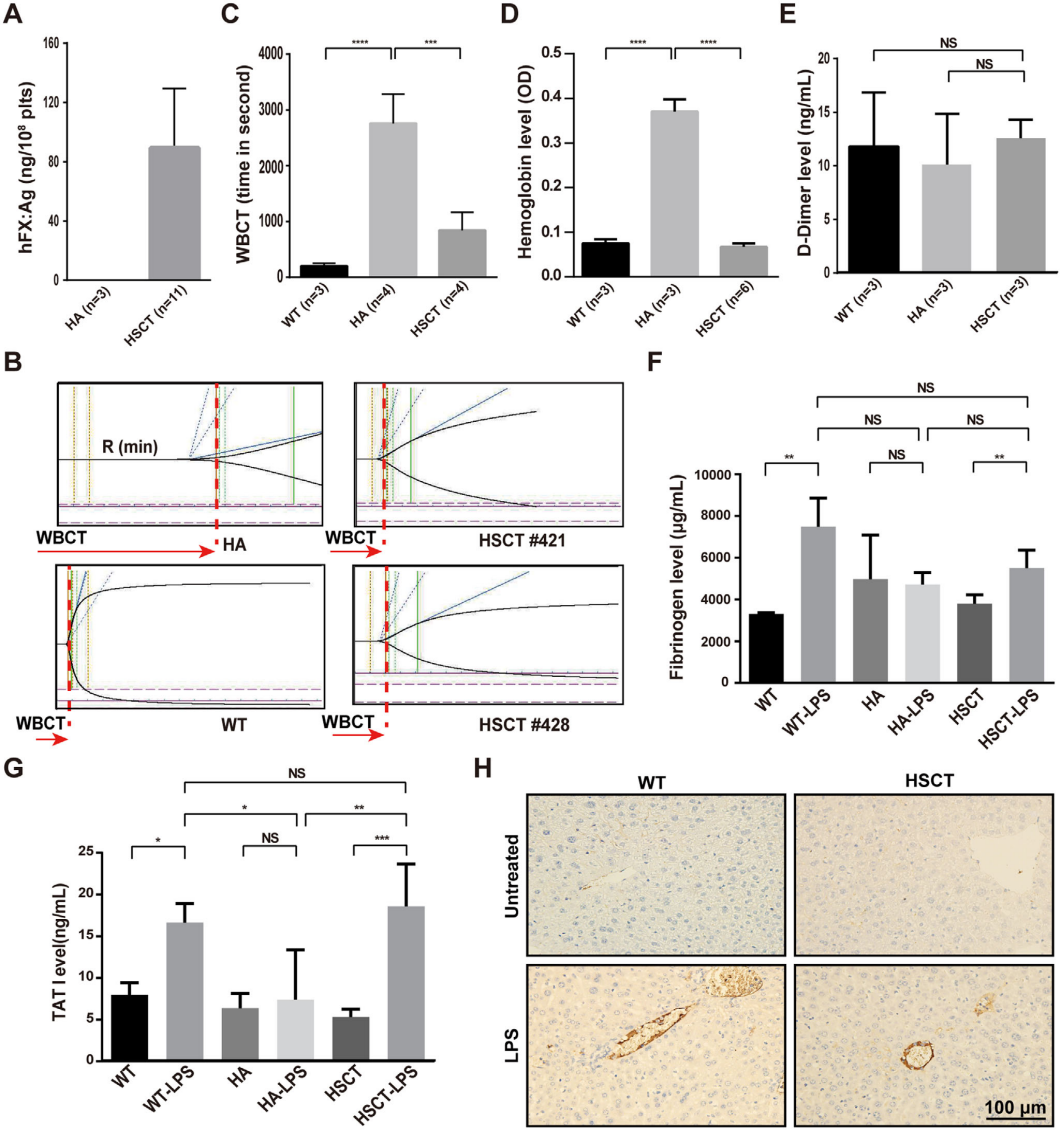
**The therapeutic effects and thrombotic risk evaluation of *2bFXa* gene therapy in HA recipient mice**. (**A**) Quantitative evaluation of hFX:Ag levels in recipient mouse platelets at week 8 after HSCT. HA, n = 3; HSCT, n = 11. (**B**) TEG analysis was performed 12 weeks after HSCT. Representative TEG traces of WT, HA, and HSCT recipients are shown, CT (clotting time) equals to R value which recorded in TEG. (**C**) WBCT determined by TEG analysis. HA and WT mice were used as controls. Mean ± SD values are shown. HA, n = 4; WT, n = 3; HSCT, n = 4. (**D**) The blood loss is expressed as the amount of hemoglobin bled from the tail into the container with normal saline. WT and HA mice were used as controls. HA, n = 3; WT, n = 3; HSCT, n = 6. (**E** to **G**) Plasma levels of D‐Dimer (**E**), fibrinogen (**F**), and TAT (**G**) were determined by ELISA. The level of these items of recipients was not significantly different from those in WT and HA mice under steady‐state condition. Compared with steady‐state mice, LPS challenge increased TAT levels 2.1‐fold and 3.5‐fold, and fibrinogen levels 2.3‐fold and 1.6‐fold, in WT and 2bFXa‐HSCT mice, respectively. HA, n = 3; WT, n = 3; HSCT, n = 6. (**H**) Fibrin deposition in the livers of the indicated mice was evaluated by immunohistochemistry. Representative images from one of three mice per group are shown. Scale bars, 100 μm. The mice were tested 8 weeks post‐transplantation. HA, hemophilia A mice. WT, S129 wild‐type mice. HSCT, 2bFXa‐HSC transplanted recipient mice. *P < .05, **P < .01, ***P < .001, and ****P *<*.0001. NS, not significant

We performed assays to evaluate the therapeutic effect of platelet‐stored FXa in HA mice. We used the TEG assay to analyze the WBCT of the mice. The hFX:Ag in recipient HA mice was 92.67 ± 38.52 ng/10^8^ platelets 8 week after HSCT (Figure 4A); the WBCT of the mice was 843 ± 279 s (n = 4), significantly shorter than that of HA controls (2762 ± 452 s, n = 4), and the average WBCT of WT mice was 206 ± 39 s in this assay (Figures 4B–4C). Then we performed the tail bleeding assay to assess blood loss of the mice after tail snip; blood loss of the HSCT recipients was close to that of WT mice and significantly lower than that of HA mice (Figure 4D). These results demonstrated amelioration of bleeding phenotype in the recipient mice, suggesting the hemostatic effect of platelet‐stored FXa.

It is well known that elevated FXa level in plasma increases the risk of thrombosis, which was a major side effect of previous attempts in treating hemophilia with FXa. In our studies, the number of platelets in recipient mice maintained in the normal range, raising thus the impossibility of increased risk of thrombosis in these mice. To address the risk of thrombosis, we measured D‐Dimer, a sensitive indicator of thrombosis, in the recipient mouse plasma. The D‐Dimer level of recipients was not significantly different from that of WT and HA mice (Figure 4E). Meanwhile, the fibrinogen and TAT levels in plasma were similar in WT, HA, and HSCT recipients (Figures 4F–4G), indicating an absence of aberrant thrombosis at the steady states in recipients. In addition, we subjected the recipient mice to a lethal lipopolysaccharide (LPS) challenge to determine whether platelet‐stored FXa could increase the thrombotic risk under inflammatory and prothrombotic conditions. After the LPS challenge, the TAT level was increased 2.1‐fold in WT mice and 3.5‐fold in HSCT mice (Figure 4F); fibrinogen level was increased 2.3‐fold in WT mice and 1.6‐fold in HSCT mice (Figure 4G). However, no significant difference was found in either TAT or fibrinogen level between WT mice and HSCT mice after LPS challenge. Fibrin deposition upon LPS challenge was found in liver, heart, kidney, and lung in both HSCT and WT mice, whereas no or little fibrin deposition was observed in the untreated control mice (Figure 4H and Figure S4A‐C). There was also no obvious difference between HSCT and WT mice, although the antibody used cannot distinguish fibrin from fibrinogen. These results tended to support the safety of platelet‐stored FXa in treated HA mice.

### 3.4 Phenotypic amelioration of HA recipient mice carrying inhibitors and sustained expression of FXa in sequential HSCT recipients

**FIGURE 5:**
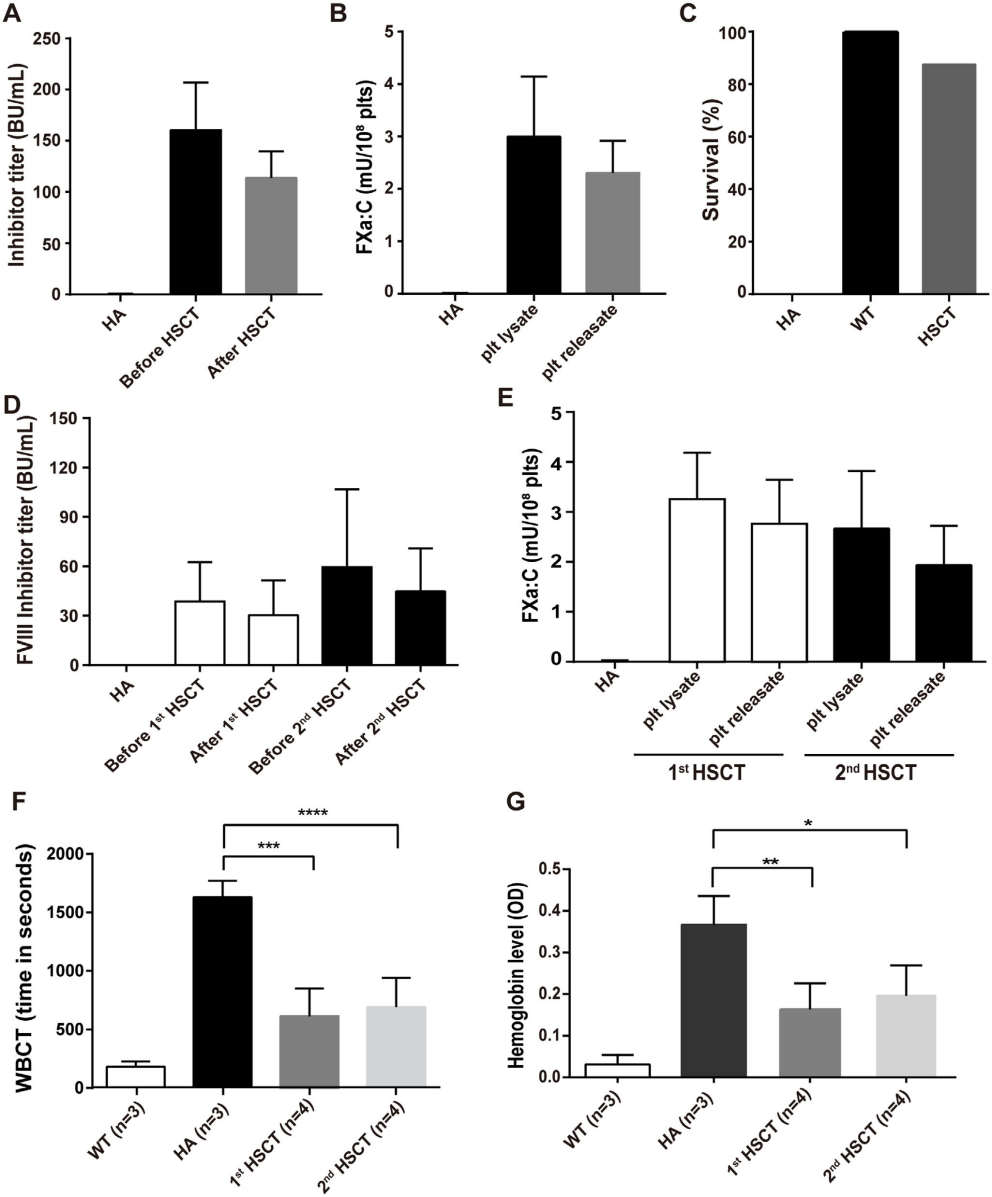
**Long‐term therapeutic effects of FXa in HA mice containing FVIII inhibitors**. (**A**) Inhibitor titres in HA mice before and after 2bFXa‐HSCT. The inhibitor titre was determined by Bethesda assay 1 week after the last immunization and 4 weeks post‐HSCT. HA, n = 4; HSCT, n = 8. (**B**) FXa:C levels in platelet lysates and releasates after platelet stimulation. Platelets were collected from HA recipient mice 4 weeks post‐transplantation. HA, n = 4; HSCT, n = 8. (**C**) The modified survival rate of HA recipient mice. Tail clipping test was performed 8 weeks post‐HSCT. HA, n = 4; WT, n = 4; HSCT, n = 8. (**D**) Inhibitor titres in HA mice before and after the 1^st^ and 2^nd^ 2bFXa‐HSCT. The inhibitor titre was determined 1 week after the last immunization and 8 weeks post‐HSCT. HA, n = 3; 1^st^ HSCT, n = 6; 2^nd^ HSCT, n = 6. (**E**) FXa:C levels in platelet lysates and releasates after platelet stimulation. Platelets were collected from 1^st^ and 2^nd^ HA recipient mice 4 weeks post‐transplantation. HA, n = 4; 1^st^ HSCT, n = 6; 2^nd^ HSCT, n = 6. (**F**) WBCT determined by whole blood TEG analysis. HA and WT mice were used as controls. Mean ± SD values are shown. HA, n = 3; WT, n = 3; 1^st^ HSCT, n = 4; 2^nd^ HSCT, n = 4. (**G**) The total blood loss is regarded as the amount of hemoglobin bled from the tail bleeding into normal saline. WT and HA mice were used as controls. HA, n = 3; WT, n = 3; 1^st^ HSCT, n = 4; 2^nd^ HSCT, n = 4. HA, hemophilia A mice. WT, S129 wild‐type mice. HSCT, 2bFXa‐HSC transplanted hemophilia A recipient mice. *P < .05, **P < .01, ***P < .001, and ****P < .0001

Encouraged by the therapeutic effect of platelet‐stored FXa in HA mice, we determined the efficacy of FXa in the presence of FVIII inhibitors. We developed FVIII inhibitors in eight HA mice by immunizing the mice with rhFVIII. All mice developed FVIII inhibitors (160.39 ± 46.69 BU/mL) after the immunization (Figure 5A). Next, we transplanted the mice with 2bFXa‐LV‐transduced Sca1^+^ cells. The blood cell counts reached the normal range 4 weeks after HSCT (Figure S5). The FXa:C levels were 2.99 ± 1.14 mU/10^8^ platelets (equivalent to 6% of normal mouse plasma FX) and 2.31 ± 0.61 mU/10^8^ platelets in platelet lysates and releasates, respectively (Figure 5B). The FVIII inhibitor titre was 113.76 ± 25.96 BU/mL (Figure 5A). The mice were tested by the tail clipping test, 7 out of 8 mice (87.5%) passed the challenge (Figure 5C), suggesting that the mice were protected from bleeding. One animal that failed to pass the challenge exhibited FXa:C of 1.06 mU/10^8^ platelets, which is the lowest level in the group. In addition, we confirmed the efficacy of platelet‐stored FXa in the tail bleeding assay. Six HA mice were immunized with rhFVIII and transplanted with 2bFXa‐LV‐transduced Sca1^+^ cells, FVIII inhibitor titres were 38.50 ± 23.99 BU/mL and 30.24 ± 21.15 BU/mL before and eight weeks after HSCT, respectively (Figure 5D). The FXa:C were 3.26 ± 0.93 mU/10^8^ platelets and 2.76 ± 0.88 mU/10^8^ platelets in platelet lysates and releasates, respectively (Figure 5E). The WBCT of the mice was 614 ± 236 s (n = 4), which was significantly shorter than that of the HA controls (1630 ± 140 s, n = 3), and the average WBCT of WT mice was 182 ± 45 s (Figure 5F). In the tail bleeding assay, the OD value of blood loss of the HSCT recipients was 0.16 ± 0.06, which was significantly lower than that of HA mice (0.37 ± 0.07) (Figure 5G). The results demonstrate that platelet‐derived FXa could alleviate the bleeding phenotype in the presence of FVIII inhibitors.

Afterwards, we did secondary transplantation to confirm genetic modification of long‐term engrafting HSCs. BM mononuclear cells were collected from some first HSCT recipients and transplanted into leathally‐irradiated rhFVIII‐immunized HA mice. FVIII inhibitor titres were 59.49 ± 47.31 BU/mL and 44.80 ± 26.05 BU/mL before and eight weeks after transplantation (Figure 5D). The FX:C were 2.66 ± 1.16 mU/10^8^ platelets and 1.94 ± 0.79 mU/10^8^ platelets in platelet lysates and releasates, respectively (Figure 5E). The *2bFXa* gene copy number was estimated at 0.42 ± 0.13 copies per cell (Figure S6). The WBCT was 690 ± 251 s (n = 4), which was significantly shorter than that of the HA controls (Figure 5F). In the tail bleeding assay, the blood loss of the recipients was 0.19 ± 0.07 (OD value), which was significantly lower than that of HA mice (Figure 5G). The results demonstrate that long‐term HSCs could be genetically modified, ensuring a long‐term therapeutic efficacy.

### 3.5 Platelet‐targeted expression of FXa in HB Mice with and without FIX inhibitors

**FIGURE 6:**
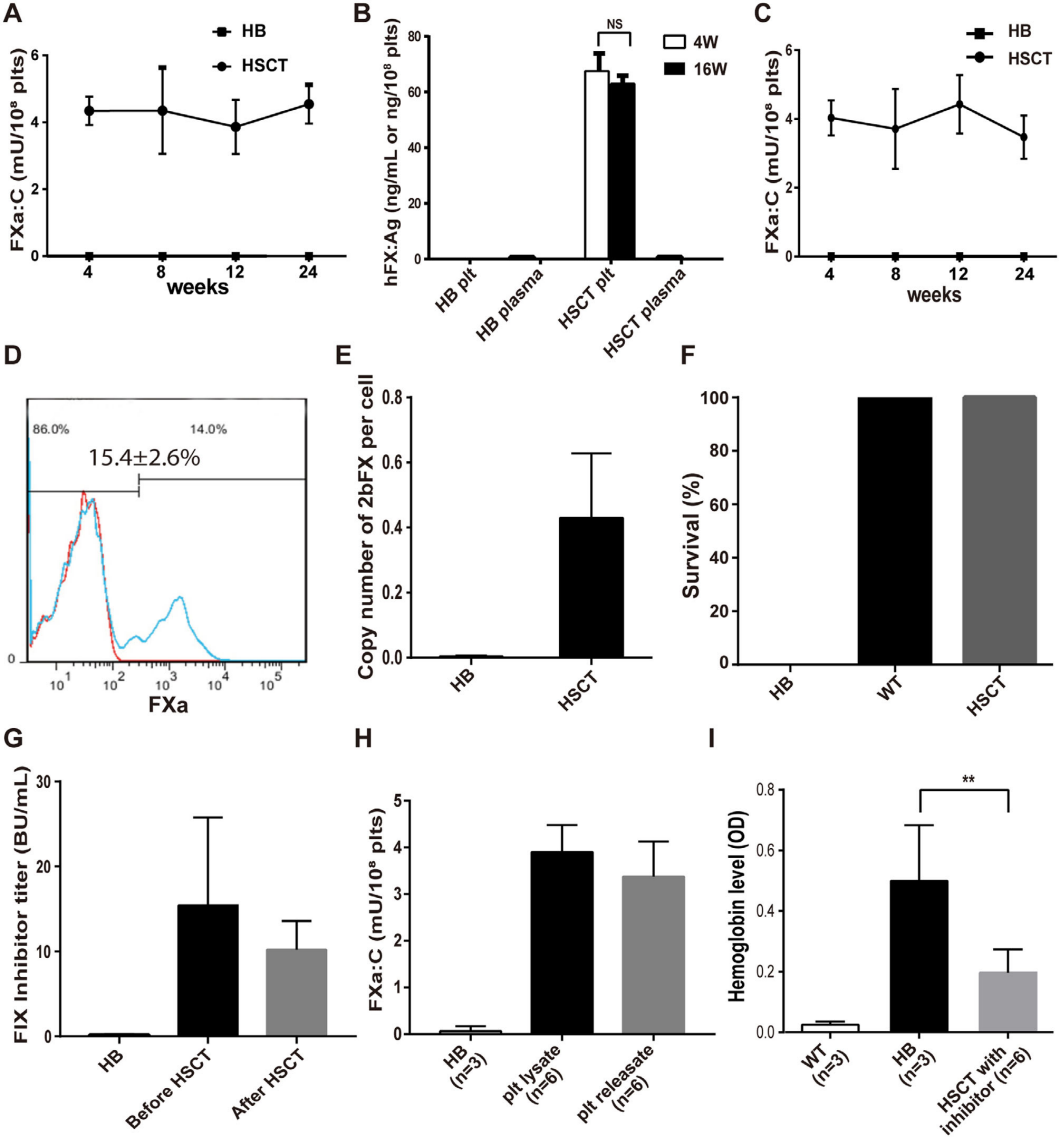
**Characterization of FXa in 2bFXa‐HSC‐transplanted HB mice with and without inhibitors**. (**A**) Quantitative evaluation of FXa:C levels in platelet lysates of the HSCT recipients. HB, n = 4; HSCT, n = 5. (**B**) Quantitative evaluation of hFXa:Ag levels in mouse plasma and platelets. Mouse plasma and platelets were collected at week 4 and week 16. HB, n = 4; HSCT, n = 5. (**C**) FXa:C level in releasates of HB recipient mice. HB, n = 4; HSCT, n = 5. (**D**) Representative results of flow cytometry analysis of HB mouse platelets. The average number indicates the level of FXa positive platelets in 5 mice. Red line, HB mouse; blue line, HSCT recipient mouse. (**E**) *2bFXa* copy number in mice 4 weeks post‐HSCT. A copy number of *2bFXa* in peripheral blood genomic DNA was measured. HB, n = 4; HSCT, n = 5. (**F**) Modified survival rate of HSCT recipient mice without inhibitors after tail clipping test. The HB mice were tested 8 weeks post‐HSCT. HB, n = 4; WT, n = 4; HSCT, n = 5. (**G**) Inhibitor titres in HB mice before and after 2bFXa‐HSCT. The inhibitor titre was determined by Bethesda assay 1 week after the last immunization and 6 weeks post‐HSCT. HB, n = 3; HSCT, n = 6. (**H**) FXa:C levels in platelet lysates and releasates after platelet stimulation. Platelets were collected from HB recipient mice with inhibitors 4 weeks post‐transplantation. HB, n = 3; HSCT, n = 6. (**I**) The blood loss is regarded as the amount of hemoglobin bled from the tail bleeding assay. WT and HB mice were used as controls. HB, n = 3; WT, n = 3; HSCT recipients with inhibitors, n = 6. HB, hemophilia B mice. WT, C57BL/6 wild‐type mice. HSCT, 2bFXa‐HSC transplanted hemophilia B recipient mice. **P < .01

To investigate if the platelet‐stored FXa could reach the same therapeutic effect for HB, we employed the same gene therapy strategy on HB mice. Lethally irradiated HB mice were transplanted with 2bFXa‐LV‐transduced Sca1^+^ cells, four weeks after HSCT, the FXa:C values were 4.34 ± 0.43 mU/10^8^ platelets (equivelant to 8.68% of normal mouse plasma FX) in platelet lysates and 4.03 ± 0.51 mU/10^8^ platelets in releasates; the levels remained constant until the study ended at week 24 (Figures 6A and 6C). The hFX:Ag was 67.46 ± 11.71 ng/10^8^ platelets in platelet lysates at week 4 and 62.94 ± 8.18 ng/10^8^ platelets at week 16, but no hFX:Ag was detected in the recipient plasma (Figure 6B). The specific activity of FXa was 64.3 ± 6.37 mU/mg and 61.3 ± 12.87 mU/mg at week 4 and week 16, respectively. About 15.4% ± 2.6% (range: 12.8%−18.6%) platelets were FXa positive in the flow cytometry analysis (Figure 6D). The *2bFXa* gene copy number in peripheral blood genomic DNA was 0.43 ± 0.19 copies per cell (Figure 6E). All blood cell counts recovered to the normal range 8 weeks after the HSCT and maintained the same levels throughout the study period (Figure S7). As predicted, all transplant recipient mice tolerated the tail clipping challenge (Figure 6F), suggesting that platelet‐stored FXa could also correct the bleeding phenotype of HB mice.

Furthermore, we determined the efficacy of platelet‐stored FXa in the presence of FIX inhibitors. We developed FIX inhibitors in the HB murine model by immunizing the mice with rhFIX. The inhibitor titre was 15.42 ± 10.84 BU/mL (Figure 6G). The mice were then transplanted with 2bFXa‐LV‐transduced Sca1^+^ cells. Six weeks after the HSCT, FXa:C were 3.90 ± 0.58 mU/10^8^ platelets in platelet lysates and 3.38 ± 0.75 mU/10^8^ platelets in releasates (Figure 6H), and the FIX inhibitor titre was 10.59 ± 3.64 BU/mL (Figure 6G). In the tail bleeding assay, the OD value of blood loss of the HSCT recipients was 0.20 ± 0.08, which was significantly lower than that of HB mice (OD value = 0.50 ± 0.18) (Figure 6I). Taken together, the results indicate that the platelet‐stored FXa was effective for alleviating the blood diathesis of HB either with or without FIX inhibitors.

## 4 DISCUSSION

Although hemophilia management based on replacement therapy can be achieved, treatment of patients with FVIII or FIX inhibitors still represents a bottleneck, because of uncertain long‐term therapeutic efficiency and heavy economic burden. The newly developed FVIII mimic agent emicizumab, which binds factor IXa and factor X to exert cofactor function of factor VIIIa, shows great effects for prophylaxtic management of HA, especially it provides a new effective treatment for HA with inhibitors.^28^, ^29^ However, emicizumab might also face the problem of neutralizing antibody against itself.^30^, ^31^ On the other hand, effective treatment of HB with inhibitors is still lacking. Hence, more efforts still need to be made to develop new treatment for hemophilia with inhibitors. Gene therapy, a treatment that has the potential to cure diseases, has brought new hope for HB and HA owing to the use of adenovirus‐associated virus (AAV) vector for FIX or FVIII gene transfer.^32^, ^33^ However, AAV‐based gene therapy might encounter a challenge when facing inhibitors. In parallel to AAV clinical trials, other approaches of hemophilia gene therapy have been performed. Studies reported that FVIII stored by platelets could effectively treat HA mice with pre‐existing FVIII inhibitors, though further increasing the FVIII level to improve therapeutic effects seemed rather difficult.^34^ However, platelet‐stored FIX was unsuccessful in HB mice with FIX inhibitors.^17^ Ohmori et al demonstrated that platelet‐expressed FVIIa could initiate blood coagulation and correct bleeding phenotype in HA mice with FVIII inhibitors, but the therapeutic efficacy of platelet‐expressed FVIIa was still low.^16^

In this work, we explored the possibility of targeting storage of FXa in platelets for the treatment of hemophilia with inhibitors, with inspiration from previous work on other bypassing agents and the recent work on the zymogen‐like FXa showing better therapeutic efficacy than FVIIa in HA and HB mice.^13^, ^35^ Two main questions were raised. First, could functional FXa be efficiently expressed in platelets via *ex vivo* gene transfer at the level of HSPC? Second, could the risk of leakage of FXa and resultant prothrombotic status be prevented?

Our results showed that *2bFXa*‐transduced HSPC, after HSCT, could differentiate toward megakaryocyte/platelet lineage and generate FXa‐containing platelets, which exerted durable therapeutic effects in HA and HB mice. It is conceivable that when FXa‐containing platelets were activated at the injury sites, FXa could be quickly released together with other components of platelets and participate in the *in situ* coagulation reaction. Therefore, the current FXa expression level is effective in ameliorating bleeding phenotype, platelet‐stored FXa could be used as a potential bypassing agent for the treatment of both HA and HB patients. Previous studies using the same strategy for FIX and FVIIa showed that FIX could be stored in α‐granules,^17^ while FVIIa in the cytoplasm of platelets.^16^ Both of FIX and FVII are vitamin K‐dependent proteins. It was thus speculated that the proteins could be properly γ‐carboxylated in megakaryocytes/platelets.^16^, ^17^, ^36^ FX is also a vitamin K‐dependent protein, and its activity is mainly determined by γ‐carboxylation for post‐translational modification.^16^, ^17^ We don't have the data of γ‐carboxylation for platelet‐derived FXa, but the results that platelet‐derived FXa was active and functional in HSCT recipient mice support this speculation. Besides, we found that FXa was co‐localized with endogenous VWF in platelet α‐granules, similar to that of the previous report of FIX. And the ratio of released FXa from activated platelets was also similar to that of platelet‐stored FIX.^17^ It has been well established that during the process of prothrombin activation, FXa and FVa form a prothrombinase complex. Since factor V is physiologically stored in platelet α‐granules, the storage of FXa in the same organelles might facilitate the binding of FXa and FVa, providing a possible therapeutic advantage. This remains to be confirmed in future studies.

How to avoid the adverse effects of platelet‐stored FXa gene therapy was another challenge. Physiologically, many endogenous FXa antagonists such as tissue factor pathway inhibitor and antithrombin III exist in plasma to inactivate FXa. FXa is cleared in a short time once it is formed in plasma. In previous studies, FXa treatment of hemophilia animals met difficulties because of prothrombotic risk. Direct infusion of FXa into the blood of hemophiliacs was unsuccessful.^11^ In the present work, we hypothesized that if FXa could be specifically expressed within platelets, it would be protected from immediate clearance. Meanwhile, platelet‐stored FXa should be released at the injury site, of which the precise local concentration of FXa remains to be determined, the exposure of FXa in plasma would be limited so that the risk of thrombosis significantly diminished. In support of this, the plasma FXa in recipient mice was undetectable, contrarily to the previous reports on platelet‐stored FIX showing the presence of a small amount of FIX in plasma.^17^, ^36^ The reason for the different behaviors of the two proteins, FXa versus FIX, is unknown. FXa's rapid clearance in plasma might explain the difference, this was supported by the result that less FXa than FX was found in the plasma of hydrodynamically injected mice. However, the possibility of leakage of a small amount of FXa from platelets cannot be ruled out by current data, further studies with increased FXa expression level may clarify this issue. Anyhow, our results demonstrate that FXa is limited in platelets under the current expression level. Besides, the level of D‐Dimer, TAT and fibrinogen, and other plasma coagulation activation markers in recipient mice was the same as those in the control animals. The HA and HB recipient mice had normal life behavior with stable body weights. In the LPS induced severe inflammation model, there was no significant difference between the HSCT recipient and WT mice in terms of coagulation activation markers. Nonetheless, caution should be taken in the interpretation of our results, as previous work showed the clot formed by platelet‐derived FVIII was unstable and pro‐embolic.^37^ In addition, the organ fibrin deposition test was done with an antibody against fibrinogen. So fibrinogen deposition cannot be excluded from the cross‐linked fibrin deposition. Future studies with sensitive technology will be performed to further address the issue of prothrombotic tendency.

The development of inhibitors against the therapeutic protein is a critical issue during hemophilia treatment, even for gene therapy, because the protein used for the treatment is the one missing from patients. FX is innately expressed in the plasma of HA or HB patients. It is reasonable to speculate that the immune response would not work against the platelet‐stored FXa. Packaging of FXa in platelets could further reduce the chance of antibody development, as already demonstrated by targeting expression of FVIII and FIX in platelets.^36^, ^38^ This may be an advantage of this gene therapy strategy over others. Furtherly, since FXa is the direct activator of prothrombin, this strategy might also be effective in the treatment of other bleeding disorders due to insufficient FXa induced by coagulation factors, such as the deficiency of factor VII and FX and the Type 3 von Willebrand disease.

In future research, the precise level of HSPC engraftment and platelet chimerism for correcting hemostasis, including the ability for the management and prophylactics of spontaneous bleeding, should be defined. The mouse with the FXa level of 1.06 mU/10^8^ platelets (equivalent to 2.12% of normal mouse plasma FX) did not passed the tail clipping challenge might give us a hint of the lower limit of effective FXa level. However, the number of mice was too small to draw a definite conclusion, further studies are needed to address this issue. A comparative study is also interesting to determine if platelet‐expressed FXa exhibits improved therapeutic effects than previously described platelet‐expressed FVIII, FIX, and FVIIa. A comparison to platelet‐store zymogen‐like FXa should be meaningful as well. These investigations could provide insight into the safety of the platelet‐targeted gene therapy strategy since these proteins are not the ones normally synthesized in platelets. Moreover, whether FXa expressed by platelets can behave the same function in hemostasis as compared to that circulated in plasma needs to be explored.

Finally, we must face the challenge as how to translate our experimental results into clinics. The HSCT protocols, which are mainly used for the treatment of hematopoietic malignancies, include myeloablative conditioning by irradiation or chemotherapy. This approach can cause substantial acute toxicity and long‐term complications in hematopoietic/immune system and non‐hematopoietic organs, constituting thus a major obstacle for its indication in less severe, nonmalignant genetic diseases like hemophilia, β‐thalassemia, and sickle‐cell anemia. Recently, it has been shown that myeloablative conditioning is not necessary for these diseases. Non‐myeloablative conditioning or non‐conditioning was demonstrated to be effective in clinical trials.^39^, ^40^ A group reported a hematopoietic‐cell‐specific internalizing immunotoxin, which could effectively and reversibly condition immunocompetent mice.^41^ A single dose of the immunotoxin, CD45–saporin (SAP), enabled efficient engraftment of donor cells (> 90%) and full correction of sickle‐cell anemia. A valine‐restricted diet was also demonstrated to be effective in facilitating HSPC engraftment.^42^ These new non‐myeloablative conditioning strategies might provide a feasible solution for hemophilia gene therapy via HSCT. With the development of in vitro generation of platelets and megakaryocytes, infusion of platelets or megakaryocytes might also be a choice for hemophilia.^43^ Future efforts using appropriate large animal models including primates and those with inhibitors should be made for pre‐clinical investigation.

## 5 CONCLUSION

Overall, our proof of principle study demonstrates that targeting FXa expression in platelets could form a releasable storage pool of FXa in platelets. The released FXa was functional in ameliorating bleeding disorder of hemophilia, even HA and HB presenting inhibitors. At the same time, no obvious risk of hypercoagulability was caused by the platelet‐stored FXa. Our results suggest that FXa expressed and delivered by platelets could be a novel choice of gene therapy strategy for hemophilia patients, especially those who have developed inhibitors. Our approach also provides a new perspective on the use of FXa in hemophilia treatment, a controlled release of FXa might be an effective form of FXa in the treatment of hemophilia containing inhibitors.

## AVAILABILITY OF DATA AND MATERIALS

The data supporting the conclusions of this article were included within the manuscript and its supplementary files.

## AUTHOR CONTRIBUTIONS

D.W., and X.S. performed research, analyzed data, and wrote the manuscript; Q.W., X.P., and Y.D. performed animal experiments; S.Y. performed protein expression research; T.Y. performed confocal analysis; Z.W. provided the hemophilia A mouse model; J.Z., and X.X. provided critical comments on the manuscript; G.Z., Z.C., and S.C. designed the study, provided research support, and wrote the manuscript.

## CONFLICT OF INTEREST

The authors declare no competing interests.

## Supporting information

SUPPORTING INFORMATION

SUPPORTING INFORMATION
