# Supplementary material for: Activated factor X targeted stored in platelets as an effective gene therapy strategy for both hemophilia A and B
Source: Clin Transl Med. 2021 Mar 24;11(3):e375. doi: 10.1002/ctm2.375 (PMC7989710; doi:10.1002/ctm2.375)
Supplement: Supplementary file 1 — SUPPORTING INFORMATION [file CTM2-11-e375-s001.docx]

**SUPPLEMENTAL INFORMATION**

**Activated factor X targeted stored in platelets as an effective gene therapy strategy for both hemophilia A and B**

Dawei Wang^1,2*^, Xiaohu Shao^1*^, Qiang Wang^1*^, Xiaohong Pan^1^, Yujun Dai^1^, Shuxian Yao^1^, Tong Yin^2^, Zhugang Wang^4^, Jiang Zhu^1^, Xiaodong Xi^1^, Zhu Chen^1,2^, Saijuan Chen^1,2^, Guowei Zhang^1,3^

^1^State Key Laboratory of Medical Genomics, Shanghai Institute of Hematology, Rui Jin Hospital Affiliated to Shanghai Jiao Tong University (SJTU) School of Medicine. Key Laboratory of Systems Biomedicine of Ministry of Education, Shanghai Center for Systems Biomedicine, SJTU, Shanghai, 200025, China.

^2^National Research Center for Translational Medicine, Ruijin Hospital Affiliated to Shanghai Jiao Tong University School of Medicine, Shanghai, 200025, China.

^3^Key Laboratory of Aging and Cancer Biology of Zhejiang Province, Hangzhou Normal University School of Medicine, Hangzhou, Zhejiang Province, 311121, China.

^4^Shanghai Research Center for Model Organisms, Shanghai, 201203, China.

^*^These authors contributed equally to this work.

To whom correspondence may be addressed. Email: gzhang@hznu.edu.cn (G.Z.) or sjchen@stn.sh.cn (S.C.).

**
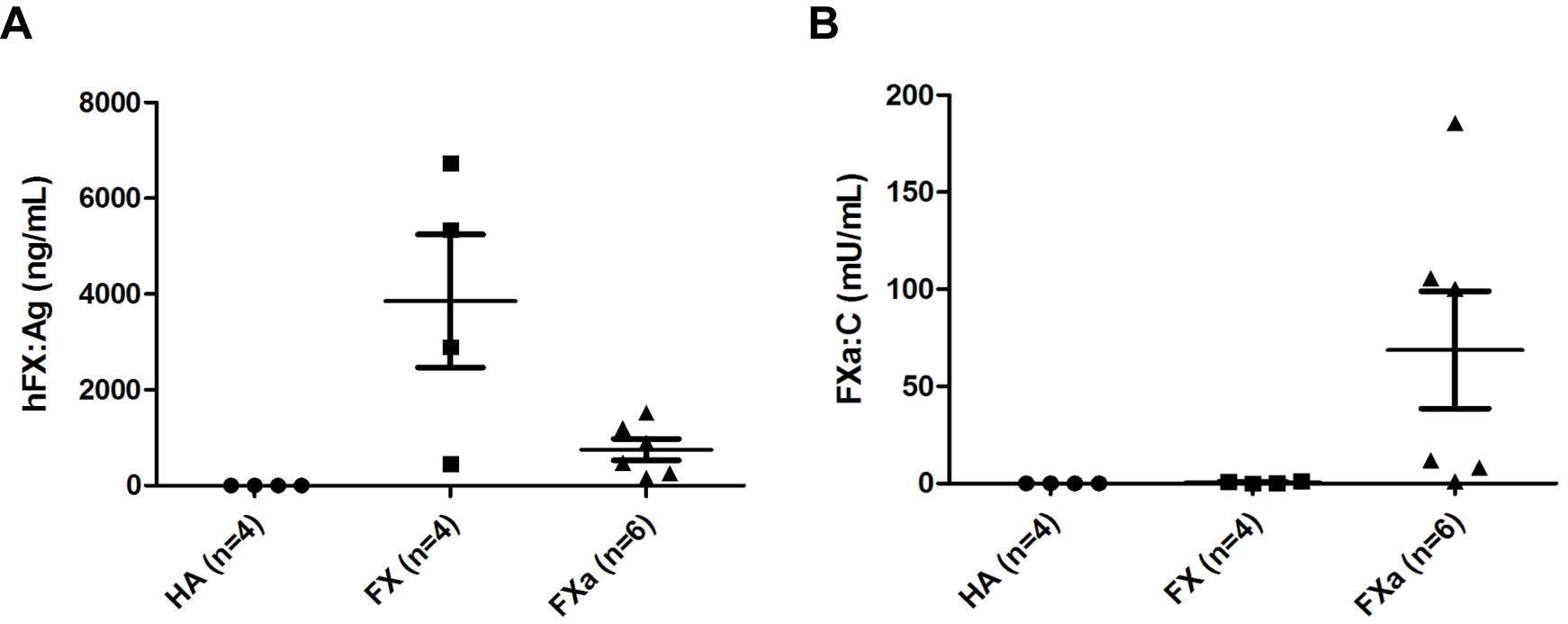
**

**Figure S1. Hydrodynamic tail vein injection of FXa cassette in HA mice.**

Plasmids PCIneoFX and PCIneoFXa were injected into HA mice, mouse plasma was collected for hFX:Ag and FXa:C quantification. (**A**) Quantitative evaluation of hFX:Ag levels in the plasma of mice receiving hydrodynamic tail vein injection. hFX:Ag was measured by a hFX specific ELISA. (**B**) Quantitative evaluation of FXa:C levels in the plasma of mice after hydrodynamic tail vein injection. FXa:C was measured by a FXa chromogenic assay. HA group, n = 4; FX group, n = 4; FXa group, n = 6. HA, hemophilia A mice.

**
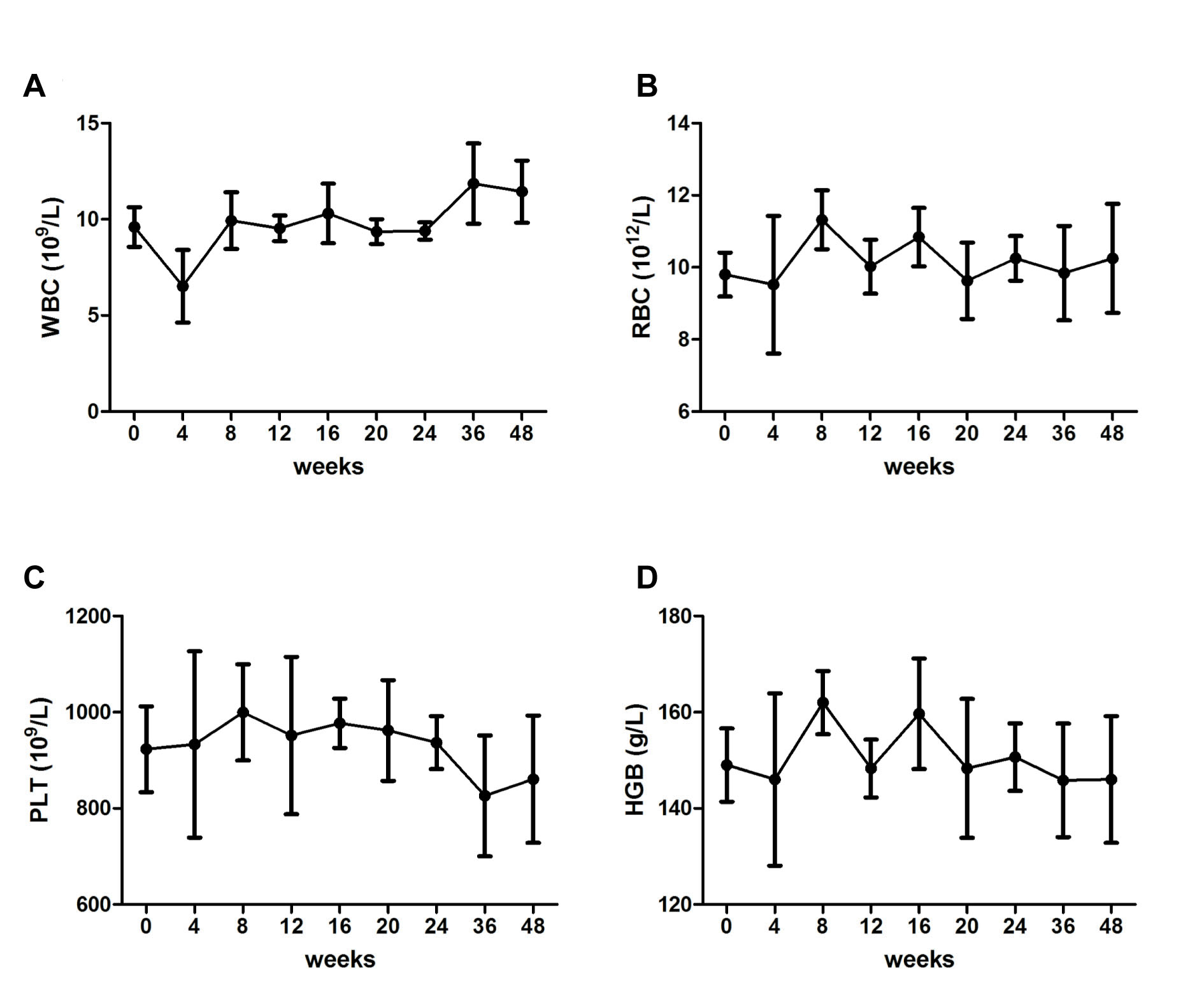
**

**Figure S2. Blood cell counts in 2bFXa-HSCT HA recipient mice** **(n = 7).** Shown are the counts for white blood cells (WBC), red blood cells (RBC), platelets (PLT), and hemoglobin (HGB).


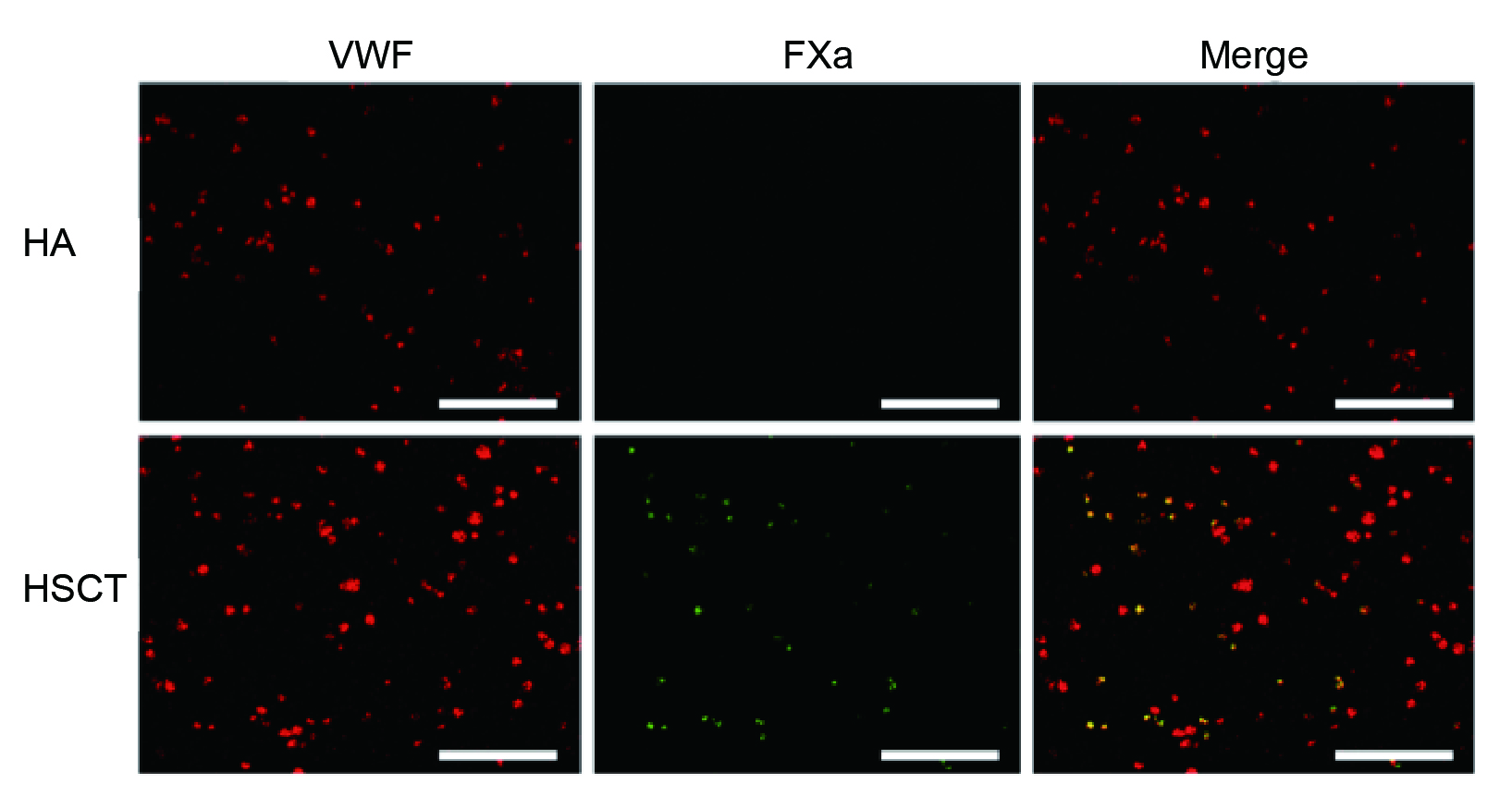


**Figure S3. Representative images of confocal microscopy analysis of FXa in platelets.** Platelets isolated from HA and transplantation recipient mice were immunostained for FXa (green) and VWF (red). The merged images show colocalization of FXa and VWF in the platelets of the HSCT recipient. Scale bars, 100 μm.

**
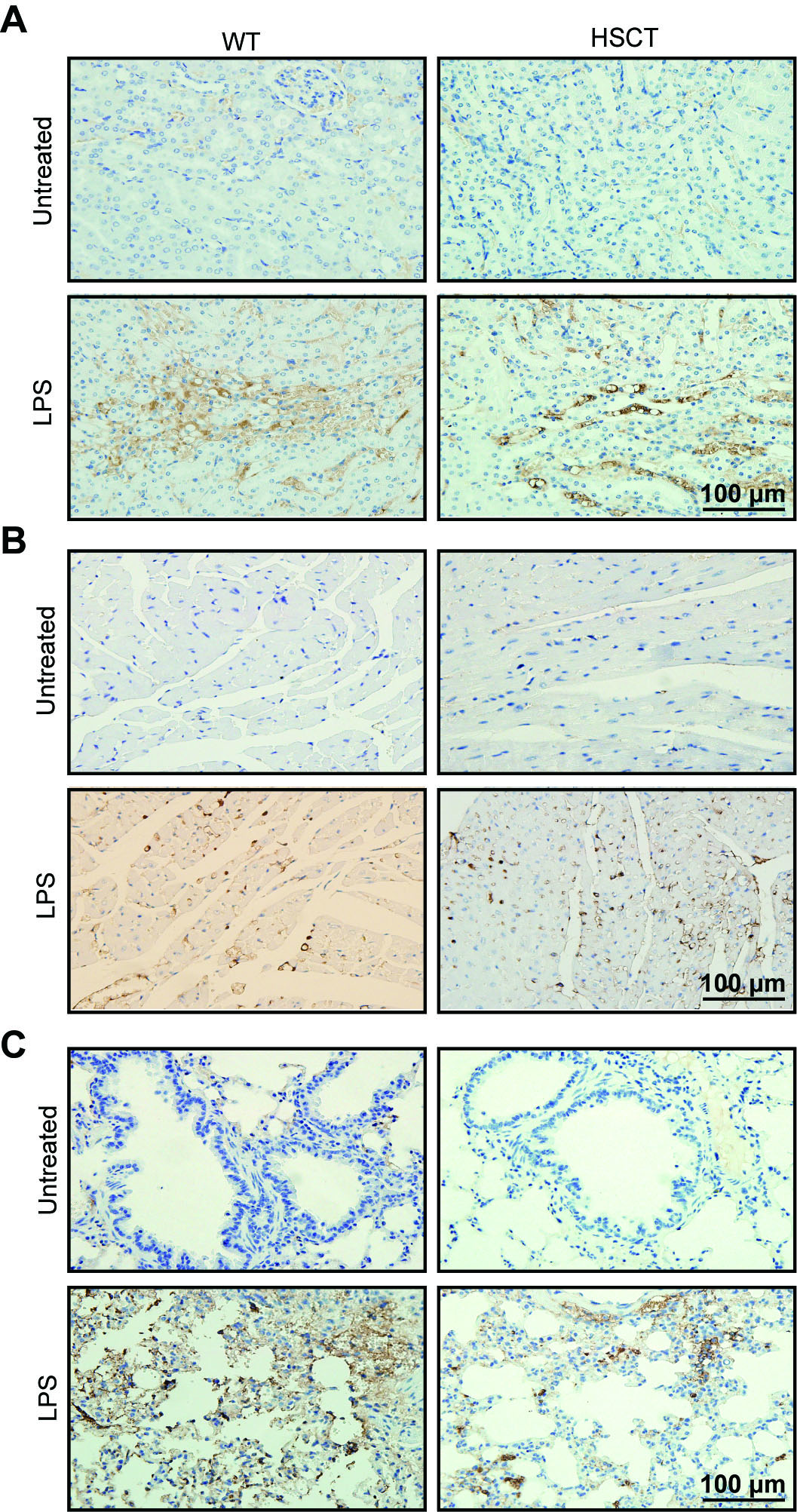
**

**Figure S4. Fibrin deposition in the organs of the HA recipients after LPS challenge by immunohistochemistry.**

(**A** to **C**) Fibrin deposition in the kidney (A), heart (B), and lung (C) of the indicated mice was evaluated by immunohistochemistry. There was no or little fibrin deposition was observed between WT and 2bFXa-HSCT recipient mice. Representative images from one of three mice per group are shown. Scale bars, 100 μm.


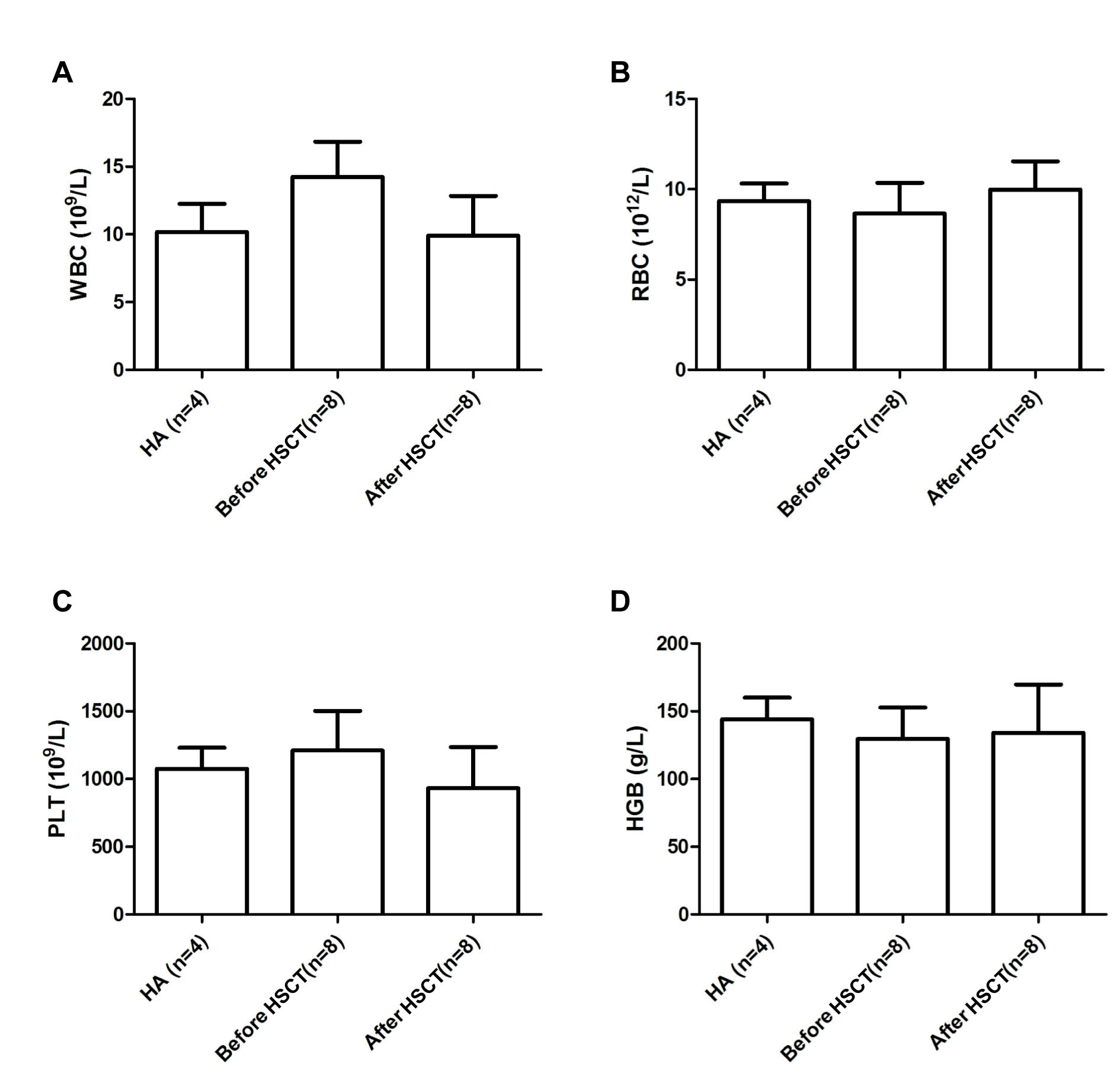


**Figure S5. Blood cell counts in 2bFXa-HSCT HA recipient mice with inhibitors.** Shown are the counts for white blood cells (WBC), red blood cells (RBC), platelets (PLT), and hemoglobin (HGB). Whole blood was collected 4 weeks post-HSCT. HA, n = 4; HSCT, n = 8.


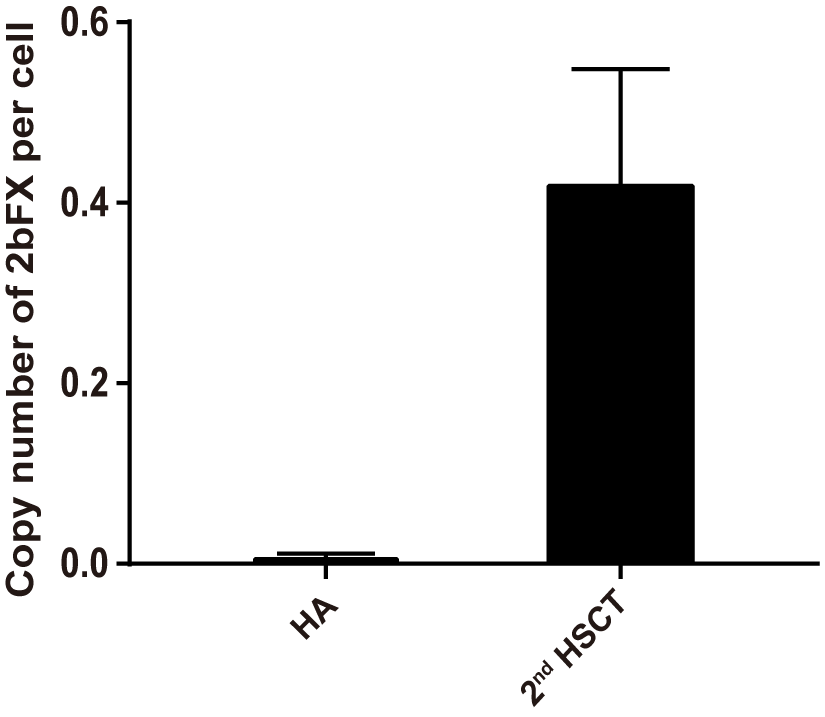


Figure S6. *2bFXa* copy number in peripheral blood of mice at 6 weeks after 2^nd^ HSCT. HA, n = 3; 2^nd^ HSCT, n = 6.


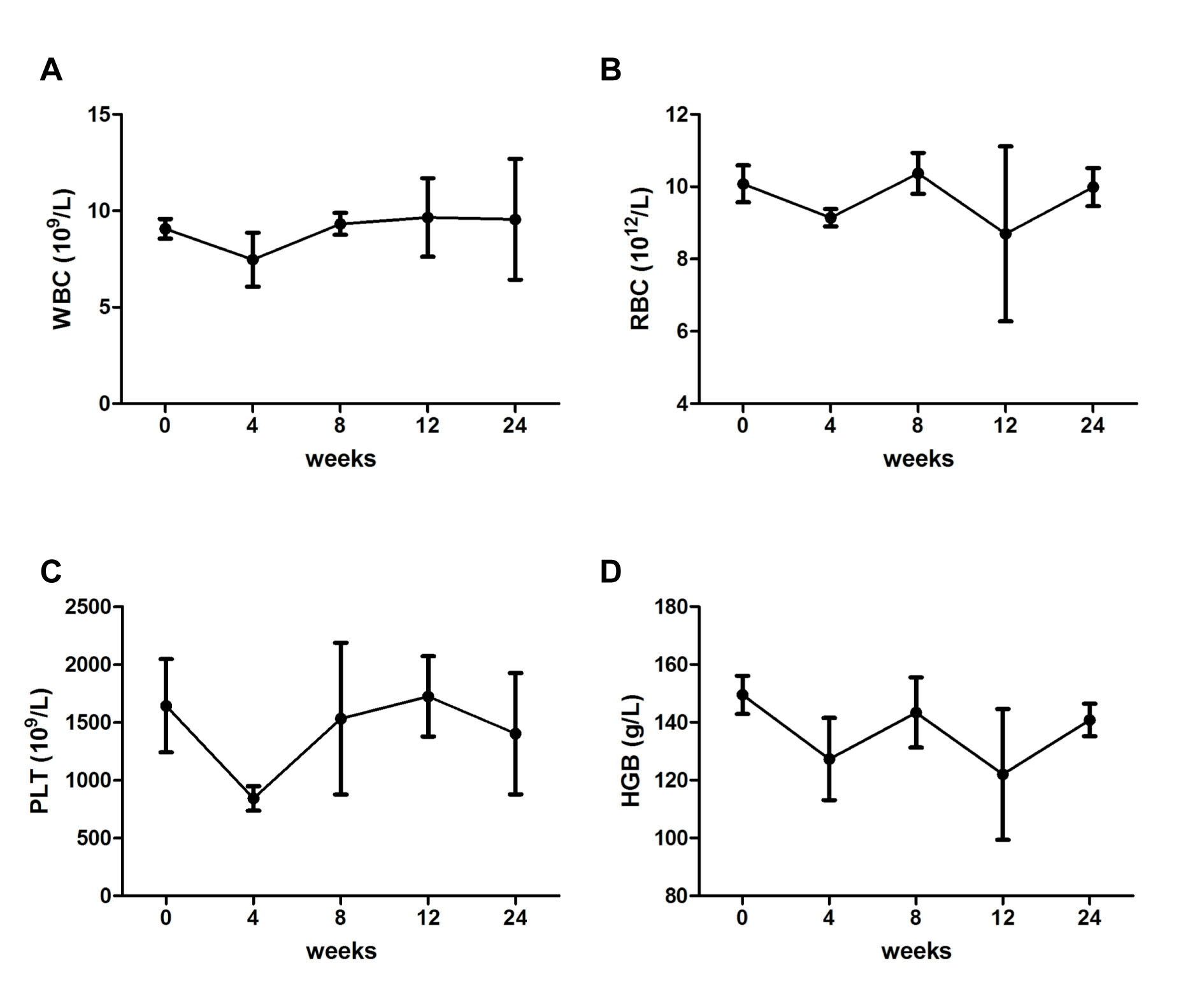


**Figure S7. Blood cell counts in 2bFXa-HSCT HB recipient mice** **(n = 5).** Shown are the counts for white blood cells (WBC), red blood cells (RBC), platelets (PLT), and hemoglobin (HGB).

**Table S1. The primers used in vector construction and real-time PCR.**

| **Name** | **Primer sequence (5’-3’)** |
| --- | --- |
| FX-F | CTCGAGAGTACTCGGCCACACCATG |
| FX-R | GTCGACGGGATCTCACTTTAATGGAGA |
| FXa-F | ACCCTGGAACGCAGGAAGAGGCGGAAAAGAATCGTGGGAGGCCAGGAATGC |
| FXa-R | GCATTCCTGGCCTCCCACGATTCTTTTCCGCCTCTTCCTGCGTTCCAGGGT |
| WRPE-F | CCGTTGTCAGGCAACGTG |
| WPRE-R | AGCTGACAGGTGGTGGCAA |
| WPRE-P | FAM-TGCTGACGCAACCCCCACTGGT-TAMRA |
| Albumin-F | TGAAACATACGTTCCCAAAGAGTTT |
| Albumin-R | CTCTCCTTCTCAGAAAGTGTGCATAT |
| Albumin-P | FAM-TGCTGAAACATTCACCTTCCATGCAGA-TAMRA |

**Video S1. Video of high-resolution confocal microscopy analysis of FXa localization in platelets.** Platelets were collected from a 2bFXa-HSC-transplanted HA recipient mouse. FXa is stained in green and VWF in red.

**Supplementary methods**

**In vitro expression of FXa**

Cells were transiently transfected with Lipofectamine 2000 (Invitrogen, Carlsbad, CA) according to the manufacturer’s instructions. 4 μg vectors pCIneoFX and pCIneoFXa, with CMV as the promoter, were transfected into 2 × 10^5^ HEK293T cells with 90% confluency, respectively. After 6 h, the medium was changed to Opti-MEM added with 4 μg/mL Vitamin K_3_ (Sigma, St. Louis, MO, USA). The cells were cultured for another 48 h and supernatant collected for further analysis. For transfection of Dami cells, 4 μg vectors pCIneo2bFX and pCIneo2bFXa were transfected into 8 × 10^6^ cells. After 6 h, the medium was changed to IMDM supplemented with 10% horse serum (Hyclone, Logan, UT, USA) and 4 μg/mL Vitamin K_3_. The cells were cultured for 24 h. The medium was then changed to Opti-MEM added with 100 nmol/L phorbol-12-myristate-13-acetate (PMA) and 4 μg/mL Vitamin K_3_ for further culturing. Cells were collected 48 h after transfection, then treated with 0.5% 3-[(3-cholamidopropyl) dimethyl-ammonio]-1-propane sulfonate (CHAPS, Sigma) on ice for 30 min. Cell lysates were collected after centrifugation at 15,000 g for 10 min.

**Western Blot analysis of FX**

The supernatant was collected 48 h after the transfection of HEK293T cells, and concentrated by Pierce® concentrator (Millipore). The samples were subjected to SDS-PAGE with or without 1 M DTT treatment. The PVDF membrane was blocked for 2 h with 5% nonfat dry milk at RT with gentle shaking. The blocked membrane was incubated with a goat anti-human factor X polyclonal antibody at 1:1,000 dilution (Affinity biologicals) at 4 °C overnight. The blot was then incubated with HRP-conjugated donkey anti-goat IgG at 1:5,000 dilution (Abcam) for 1 h in a shaker, then exposed to Immobilon^TM^ Western Chemiluminescent Substrate (Millipore) for detection.

**Blood collection and platelet activation**

Mouse plasma was collected from the tail vein or retro-orbital puncture by bleeding 90 μL blood into a tube containing 10 μL 3.8% sodium citrate and centrifuged at 1,000 g for 20 min to remove cells. Mouse serum was collected from the tail vein by bleeding approximately 100 μL blood based on a standard protocol.^1^ Briefly, blood was collected in a tube without anticoagulant. Then the tube was incubated for 1 h at 37°C and flicked with several times. After that, the tube was transferred to 4°C for another 2 h, and centrifuged at 10,000 g for 10 min for acquiring the serum. Platelet collection, lysate preparation, and activation were performed as previously described.^2^ Briefly, 100 μL mouse blood was bled into a collection tube containing 600 μL Tyrode buffer including 0.38% sodium citrate and 50 ng/mL of prostaglandin E1 (Sigma). Platelet-containing supernatant was transferred into a new tube after centrifuging at 200 g for 3 min. Platelet pellets were collected after spinning at 900 g for 20 min. For platelet lysates, platelets were treated with 0.5% CHAPS on ice for 10 min and centrifuged at 15,000 rpm for 10 min. For platelet activation, platelets were resuspended in 1 mL Tyrode buffer and washed once. Then platelets were suspended in Tyrode buffer containing 1 mM CaCl_2_, 2 μM ADP (Amresco, Solon, OH, USA), 25 μM of epinephrine (Sigma) and 25 μM of murine thrombin receptor activation peptide (GYPGKF-NH_2_, Shanghai Science Peptide Biological Technology, Shanghai, China). The mixture was incubated at room temperature (RT) for 30 min, and then releasate was collected by centrifuging at 15,000 rpm for 10 min.

**Hydrodynamic tail vein injection of HA mice**

Plasmids pCIneoFX and pCIneoFXa were extracted with Endofree Plasmid Mega kit (QIAGEN, Hilden, Germany). A total of 200 μg of plasmids in 2 mL of 0.9% NaCl solution was injected into the tail vein of HA mice within 6−8 s.^3^ After 24 h, the plasma was collected and subjected to hFX:Ag and FXa:C assays.

**References**

1. Greenfield EA. Sampling and Preparation of Mouse and Rat Serum. *Cold Spring Harb Protoc.* 2017;2017(11):pdb.prot100271.

2. Shi Q, Wilcox DA, Fahs SA, et al. Factor VIII ectopically targeted to platelets is therapeutic in hemophilia A with high-titer inhibitory antibodies. *J Clin Invest.* 2006;116(7):1974-1982.

3. Liu F, Song Y, Liu D. Hydrodynamics-based transfection in animals by systemic administration of plasmid DNA. *Gene Ther.* 1999;6(7):1258-1266.
